# Phosphoproteomics reveals malaria parasite Protein Kinase G as a signalling hub regulating egress and invasion

**DOI:** 10.1038/ncomms8285

**Published:** 2015-07-07

**Authors:** Mahmood M. Alam, Lev Solyakov, Andrew R. Bottrill, Christian Flueck, Faiza A. Siddiqui, Shailja Singh, Sharad Mistry, Maria Viskaduraki, Kate Lee, Christine S. Hopp, Chetan E. Chitnis, Christian Doerig, Robert W. Moon, Judith L. Green, Anthony A. Holder, David A. Baker, Andrew B. Tobin

**Affiliations:** 1 MRC Toxicology Unit, University of Leicester, Hodgkin Building, Lancaster Road, Leicester LE1 9HN, UK; 2 PNACL, Core Biotechnology Services, University of Leicester, Hodgkin Building, Lancaster Road, Leicester LE1 9HN, UK; 3 Faculty of Infectious and Tropical Diseases, London School of Hygiene & Tropical Medicine, Keppel Street, London WC1E 7HT, UK; 4 International Centre for Genetic Engineering and Biotechnology (ICGEB), Aruna Asaf Ali Marg, New Delhi 110 067, India; 5 Bioinformatics and Biostatistics Support Hub (B/BASH), Core Biotechnology Services, Maurice Shock Building, University of Leicester, University Road, Leicester LE1 9HN, UK; 6 Department of Microbiology, Monash University, Building 76, Clayton, Victoria 3800, Australia; 7 Division of Parasitology, MRC National Institute for Medical Research, Ridgeway, Mill Hill, London NW7 1AA, UK

## Abstract

Our understanding of the key phosphorylation-dependent signalling pathways in the human malaria parasite, *Plasmodium falciparum*, remains rudimentary. Here we address this issue for the essential cGMP-dependent protein kinase, PfPKG. By employing chemical and genetic tools in combination with quantitative global phosphoproteomics, we identify the phosphorylation sites on 69 proteins that are direct or indirect cellular targets for PfPKG. These PfPKG targets include proteins involved in cell signalling, proteolysis, gene regulation, protein export and ion and protein transport, indicating that cGMP/PfPKG acts as a signalling hub that plays a central role in a number of core parasite processes. We also show that PfPKG activity is required for parasite invasion. This correlates with the finding that the calcium-dependent protein kinase, PfCDPK1, is phosphorylated by PfPKG, as are components of the actomyosin complex, providing mechanistic insight into the essential role of PfPKG in parasite egress and invasion.

## Abstract (web summary)

Protein kinases expressed by the malaria parasite *Plasmodium falciparum* represent potentially valuable drug targets. Alam *et al*. identify proteins whose phosphorylation is dependent on the kinase *Pf*PKG, revealing a regulatory network controlling parasite egress from red blood cells and calcium signalling.

## Graphical abstract

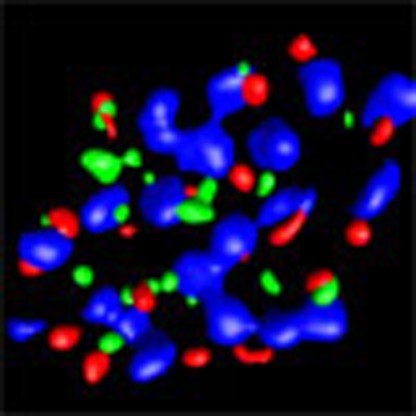

It is well established that protein phosphorylation is the primary regulatory mechanism employed by eukaryotic cells in the control of cellular processes^1^. This is clearly understood in mammalian cells where many decades of research have revealed the organization of the >500 mammalian protein kinases into phospho-signalling modules that are responsible for generating the tens of thousands of cellular phosphorylation sites^2^,^3^. These proteins are phosphorylated in a complex and often highly dynamic manner, allowing for the precise co-ordination of cellular events. Understanding the relationship between protein kinases, the nature of phospho-signalling cascades and the protein substrates associated with phospho-signalling modules is central to acquiring a full appreciation of the mechanisms that regulate cellular activity and to reveal important disease mechanisms and potential therapeutic targets.

Whereas this area has been very fruitful in mammalian cell biology, particularly when applied to the field of cancer research^4^, the understanding of phospho-signalling in malaria parasites is still in its infancy^5^. Recently, published global phosphoproteomic studies conducted on the most virulent species of human malaria parasites, *Plasmodium falciparum*, have revealed that the 80–100 parasite protein kinases^6^,^7^,^8^ phosphorylate proteins involved in nearly every aspect of the asexual blood stage of the parasite life cycle^9^,^10^,^11^,^12^. Further, studies conducted largely on the mouse malaria parasite, *Plasmodium berghei*, have revealed the importance of protein phosphorylation in the mosquito stages^13^. Hence, the essential role played by phosphorylation in the development of both asexual and sexual stages of malaria parasites, together with the high degree of phylogenetic distance between the human and malaria protein kinases, supports the notion that targeting parasite protein kinases would present therapeutic opportunities not only for the treatment of malaria but also in preventing onward transmission^5^.

One of the barriers to exploiting these opportunities is the paucity of information regarding the role of essential protein kinases and the nature of phospho-signalling cascades in *Plasmodium* parasites^11^. Here, we address these issues by combining chemical genetics and global phospho-proteomic approaches to reveal the phosphorylation events mediated by the *P. falciparum* guanosine 3′,5′-cyclic monophosphate (cGMP)-dependent protein kinase, *Pf*PKG (PF3D7_1436600). This protein kinase is known to play an essential role not only in asexual blood stages, where it is involved in both late stage schizogony^14^ and egress of the parasite from red blood cells (RBCs)^15^,^16^, but also in the mosquito stages, with demonstrated roles in gametogenesis^17^ and ookinete motility^18^,^19^ as well as late liver stage development^20^. Importantly, the action of *Pf*PKG is often associated with calcium-dependent signalling. Thus, *Pf*PKG has been reported to act upstream of the calcium-dependent protein kinase *Pf*CDPK5 to regulate parasite egress^16^. Furthermore, *Pf*PKG is reported to be important in maintaining elevated calcium levels, possibly through the regulation of phosphoinositide biosynthesis, necessary for ookinete motility in *P. berghei* and blood stage schizogony in *P. falciparum*^18^.

Our study aimed to provide an extensive evaluation of the action of *Pf*PKG in *P. falciparum* by employing a selective inhibitor, termed Compound 2 (4-[7-[(dimethylamino)-methyl]-2-(4-fluorphenyl)imidazo[1,2-*α*]pyridine-3-yl] pyrimidin-2-amine)^21^, which acts by gaining access to a hydrophobic pocket adjacent to the kinase ATP-binding pocket. The binding of Compound 2 to *Pf*PKG is thought to rely on the unusually small, threonine, gate-keeper residue^22^. Binding of Compound 2 is blocked by the substitution of the threonine gate-keeper with a bulkier glutamine residue. This mutant kinase termed *Pf*PKG_T618Q,_ has previously been demonstrated to have a reduced sensitivity for Compound 2 of >2,000-fold^17^. A mutant parasite strain, where the wild-type *Pfpkg* allele was replaced by *Pf*PKG_T618Q_, has previously been used by us^15^,^17^,^18^, and the equivalent in coccidian parasites has been used by others^21^, as a chemical genetic tool to investigate the role of PKG in malaria and other apicomplexans. Furthermore, preliminary studies from our laboratories using metabolically ([^32^P]-orthophosphate) labelled parasites indicated that *Pf*PKG may have a number of cellular substrates, although the identity and biological significance of these were not determined^23^.

Here we establish *Pf*PKG-dependent phosphorylation events in late *P. falciparum* blood stage schizonts by quantitatively comparing the changes in global phosphorylation following administration of Compound 2 to wild-type and *Pf*PKG_T618Q_ mutant parasites. These studies revealed a number of novel *Pf*PKG-dependent phospho-proteins. Most notable were those that have an involvement in parasite egress, invasion and calcium signalling.

## Results

### Identity of *Pf*PKG-dependent phosphorylation events

**Figure 1:**
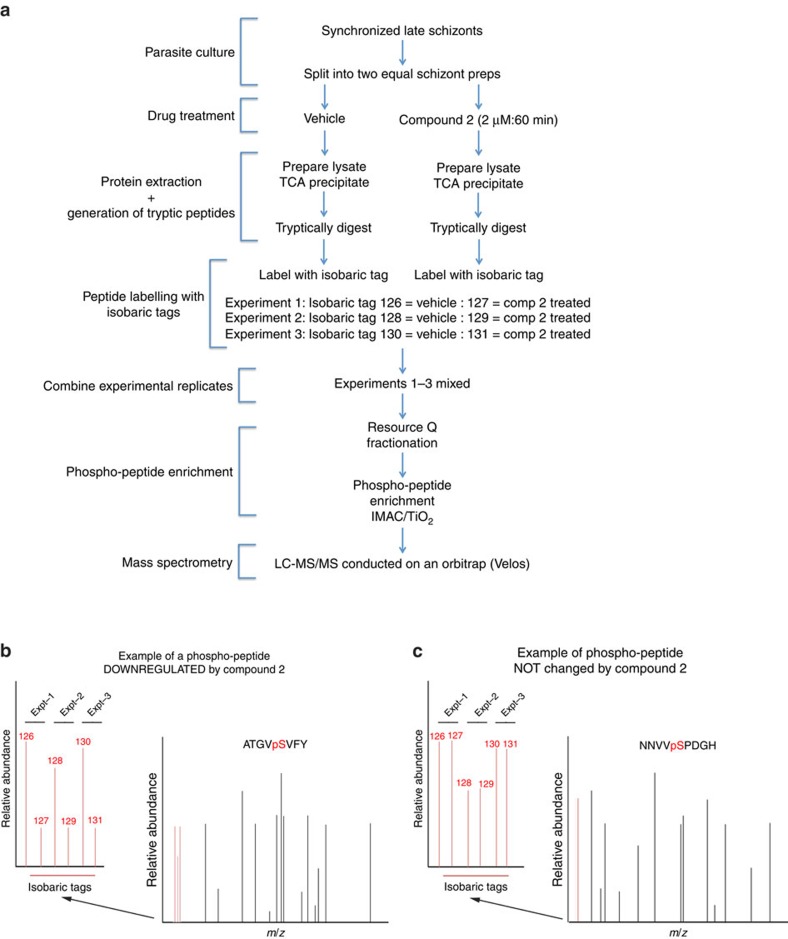
A schematic representation of the quantitative global phosphoproteomic study to determine the cellular targets for *Pf*PKG. (**a**) Late schizont stage parasites were purified on MACS column and incubated with or without Compound 2 (2 μM, 60 min) after which parasite lysates were prepared and digested with trypsin. Peptide samples from individual experiments were labelled with isobaric mass tags and the peptides from three independent experiments were pooled, then phosphopeptides were enriched by sequential fractionation using anion-exchange, PHOS-select (IMAC) and TiO_2_ beads before being analysed by LC-MS/MS. (**b**,**c**) Illustrative mass spectra showing an example of a phosphopeptide that was less abundant following Compound 2 treatment (**b**) and one that was unchanged by Compound 2 treatment (**c**).

The major challenge with using inhibitors of a specific protein kinase is differentiating what are the on- and off-target actions of the inhibitor. Here we use a genetically modified parasite strain expressing the Compound 2-insensitive *Pf*PKG mutant, *Pf*PKG_T618Q_, to define the on-target action of the inhibitor of *Pf*PKG, Compound 2. In these experiments, the targets for *Pf*PKG were identified by comparing the changes in the global phosphoproteome of wild-type late schizont stage parasites following Compound 2 treatment (2 μM, 60 min) with those changes observed following Compound 2 treatment of the *Pf*PKG_T618Q_ mutant parasite strain (Fig. 1). For a particular phosphorylation event to be considered as being mediated by *Pf*PKG (that is, on-target action of Compound 2), that particular phospho-peptide should be significantly downregulated by Compound 2 in wild-type parasites but not in *Pf*PKG_T618Q_ mutant parasites, in all biological replicates (Fig. 1). In contrast, those phospho-peptides that are downregulated equally by Compound 2 in wild-type and mutant parasites are likely due to Compound 2 acting on a protein kinase other than *Pf*PKG and therefore considered as off-target (for an example see Supplementary Fig. 1).

**Figure 2:**
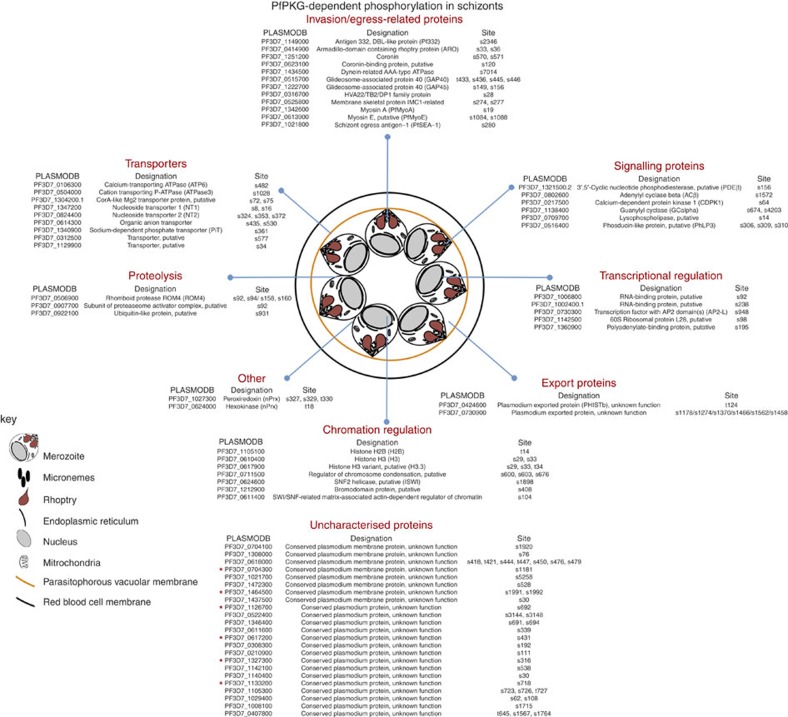
Cellular targets for *Pf*PKG in schizonts. By comparing the impact on the phosphoproteome of the *Pf*PKG inhibitor, Compound 2, in wild-type 3D7 parasites and a mutant parasite strain expressing the Compound 2 insensitive *Pf*PKG_T618Q_, we identified 107 phosphorylation sites on 69 parasite proteins as cellular targets of *Pf*PKG. The proteins were divided into Gene Ontology groups. Those marked with an * are proteins that share an association with the motor complex, suggesting that they might function as motor-associated proteins.

Here we conducted three independent experiments testing the effects of Compound 2 on wild-type and *Pf*PKG_T618Q_ mutant parasites. By applying the criteria described above, we established that of the 2,931 unique phosphorylation sites identified in wild-type schizonts (Supplementary Data 1) and 2,823 unique sites in *Pf*PKG_T618Q_ mutant schizonts (Supplementary Data 2), 107 phosphorylation sites present on 69 phospho-proteins were dependent on *Pf*PKG activity and therefore can be considered as cellular targets for *Pf*PKG (Fig. 2 and Supplementary Data 3). The mass spectrometry proteomics data have been deposited to the ProteomeXchange Consortium via the PRIDE partner repository with the dataset identifier PXD002266 and 10.6019/PXD002266.

### *Pf*PKG cellular targets

Among the cellular targets for *Pf*PKG are proteins involved in cell signalling, proteolysis, ion transport, transcriptional regulation, protein export and chromatin regulation (Fig. 2). These phosphorylation events are in addition to the *Pf*PKG-dependent phosphorylation of 23 uncharacterized proteins (Fig. 2), indicating the potential for *Pf*PKG to play a wide-ranging role in the regulation of numerous blood stage processes (Fig. 2), similar to the extended role described for PKG isoforms in mammalian cellular activity^24^. These varied cellular *Pf*PKG-targets correspond to the broad subcellular distribution of *Pf*PKG where the kinase was found at both membrane and cytoplasmic sites as well as associated with the endoplasmic reticulum^23^.

We addressed the question of how many of the 107 *Pf*PKG-dependent phosphorylation sites might be direct substrates for *Pf*PKG by assessing the number of sites that conformed to the established PKG consensus motif (K/RR/KxpS/pT)^24^. In addition, we considered the possibility that *Pf*PKG might also phosphorylate at the cAMP-dependent protein kinase (PKA) consensus site K/RxxpS/pT^9^. This is based on studies, using peptides and cellular protein substrates, that have reported extensive overlap in the primary amino-acid sequences phosphorylated by PKA and PKG^25^,^26^,^27^. Supplementary Data 4 shows that 46 of the 107 *Pf*PKG-dependent phosphorylation sites identified here conformed to one or other of these potential PKG consensus motifs, raising the possibility that these sites are directly phosphorylated by *Pf*PKG. It is, however, not uncommon for protein kinases to phosphorylate sites that do not conform to consensus sequences. Thus, the number of sites actually phosphorylated by *Pf*PKG may be higher, or indeed lower, than this estimate. What is certain is that our study demonstrates that *Pf*PKG can regulate the phosphorylation status of 69 parasite proteins either directly or indirectly via the regulation of other protein kinases, and in this way, impact on a number of parasite systems.

One such system appears to be a potential cyclic nucleotide feed-back loop. This is indicated by the fact that one of the four 3′,5′-cyclic nucleotide phosphodiesterases (PDEβ: PF3D7_1321500.2) and one of the two guanylyl cyclases (GCα: PF3D7_1138400) were phosphorylated in a *Pf*PKG-dependent manner, indicative of feed-back mechanisms operating in cGMP signalling, whereby both synthesis and degradation of cGMP might be regulated by *Pf*PKG. Furthermore, cross-talk between cGMP and cAMP signalling was indicated by *Pf*PKG-dependent phosphorylation of adenylyl cyclase (ACβ: PF3D7_0802600) on S1572 (Fig. 2). These phosphorylation events point to the possibility that cyclic nucleotide signalling in malaria parasites show complex feed-back regulation similar to mechanisms in operation in mammalian cells where PKA- and PKG-mediated phosphorylation can regulate the activity of both cyclases and phosphodiesterases^24^.

A number of proteins involved in transcription, translation and chromatin dynamics were phosphorylated in a *Pf*PKG-dependent manner, indicating that cGMP signalling might play a role in the regulation of gene expression across different levels in *Plasmodium* schizonts (Fig. 2), either through direct *Pf*PKG activity or through down-stream *Pf*PKG-regulated kinases (or phosphatases). These phosphorylation events included a number of sites on histones. One particular set of sites serine-29 (S29) and serine-33 (S33) on histone 3 (H3.1: PF3D7_0610400) contained within the peptide K**S**API**S**AGIK (underlined/bold residues indicate the phosphorylation sites) were phosphorylated in a *Pf*PKG-dependent manner, as were the equivalent sites on the histone 3 variant (H3.3: PF3D7_0617900; K**S**APV**ST**GIK). Histone-H3.1 S29 phosphorylation (H3S29) is a conserved histone modification, which in mammalian cells is mediated by Aurora B kinase during mitosis coinciding with chromosome condensation^28^. Three Aurora kinase homologues are present in the *P. falciparum* genome^29^ and histone-H3.1 peptides phosphorylated at S29 and S33 have been identified in a previous phosphoproteomic study of *Plasmodium falciparum* schizonts^12^. Moreover, the histone reader *Pf*14-3-3 has been shown to specifically bind histones H3.1 and H3.3 phosphorylated at S29 or dually phosphorylated at S29 and S33 (ref. 30). The functional significance of these histone H3 phosphorylations remains uncertain, but their high abundance suggests a global function in chromatin dynamics rather than transcriptional regulation of a small subset of genes. Similarly, histone H2B (PF3D7_1105100) was phosphorylated in a *Pf*PKG-dependent manner on threonine-14 (T14) (TG**T**GPDGK). To our knowledge, this modification has not been reported previously and is not conserved in higher eukaryotes. Interestingly, like H3.1 phosphorylation at S29, H2B phosphorylation at T14 has a proline in the +2 position, a major determinant for 14–3–3 binding^31^. Intriguingly, our phosphoproteomic analysis also revealed three *Pf*PKG-dependent phosphorylation events in a putative regulator of chromosome condensation (PF3D7_0711500), further linking *Pf*PKG activity to chromatin biology.

**Figure 3:**
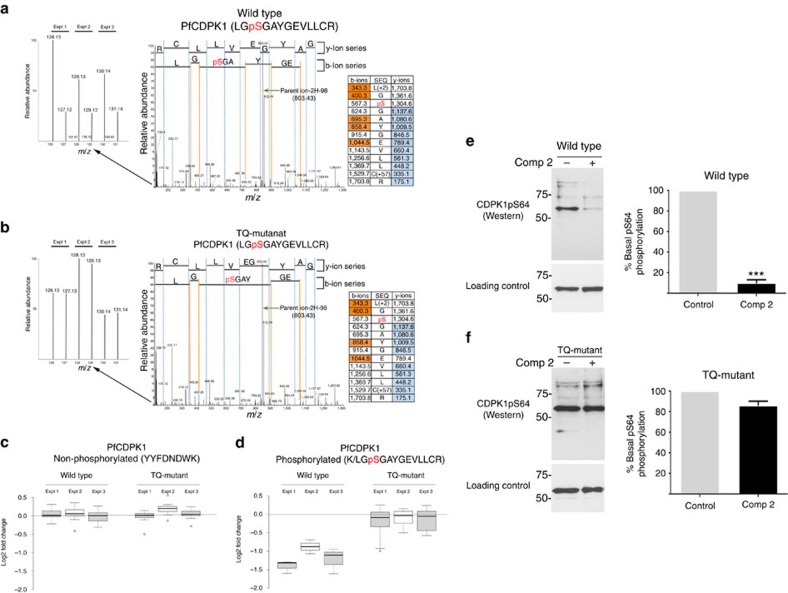
*Pf*CDPK1 is a *Pf*PKG cellular target. (**a,b**) Representative spectra from the global phosphoproteomic data demonstrating that phosphorylation of serine-64 in *Pf*CDPK1 is downregulated following Compound 2 treatment (2 μM, 60 min; Comp 2) of wild-type schizonts (**a**) but not in *Pf*PKG_T618Q_ mutant parasites (**b**). The inset on the left is a zoom of each spectrum to show the relative abundance of reporter ions from three experiments (126 and 127 from Expt. 1, 128 and 129 from Expt. 2, and 130 and 131 from Expt. 3). The inset on the right hand side is the fragmentation table showing the observed b-ions in orange and y-ions in blue. (**c**) Box plot representation of the quantitative changes in abundance of a non-phosphorylated *Pf*CDPK1 peptide derived from wild-type and *Pf*PKG_T618Q_ mutant parasites and (**d**) of peptides spanning the serine-64 phosphorylation site in *Pf*CDPK1. (**e,f**) Representative western blots and quantification of *n*=5 experiments ±s.e.m. showing changes in phosphorylation at serine-64 of *Pf*CDPK1 in wild-type parasites (**e**) and *Pf*PKG_T618Q_ parasites (**f**) using an in-house antibody to phosphoserine-64 on CDPK1 (CDPK1-pS64). Blots were stripped and re-blotted using a structural *Pf*CDPK1 antibody as a loading control. Student's paired *t*-test was applied to test statistical significance ***P<0.001.

Previous studies have also linked cGMP with calcium signalling in malaria parasites^16^,^18^. It is therefore of considerable interest that the calcium-dependent protein kinase, *Pf*CDPK1, was identified here as a cellular target for *Pf*PKG (Fig. 3 and Supplementary Data set 3). Treatment of wild-type parasites with Compound 2 resulted in a significant reduction in the phosphorylation of serine 64 (S64) within domain I of the *Pf*CDPK1 kinase domain (Fig. 3a–d). This change in phosphorylation was not due to a change in the expression in *Pf*CDPK1, as non-phosphorylated peptides (for example, YYFDFNDWK (Fig. 3c)) showed no quantitative change following Compound 2 treatment. Importantly, in the *Pf*PKG_T618Q_ mutant parasite line no change in the phosphorylation of S64 on *Pf*CDPK1 was observed in response to Compound 2 treatment (Fig. 3a–d). S64 phosphorylation on *Pf*CDPK1 can thus be considered as a prime example of an on-target action of Compound 2 on *Pf*PKG.

To further confirm these findings, parasites expressing a haemagglutinin (HA)-tagged *Pf*CDPK1, were used to probe the phosphorylation status of *Pf*CPDK1. Metabolic labelling of the *Pf*CDPK1-HA-expressing parasites followed by treatment with vehicle or Compound 2 (2 μM, 60 min) demonstrated that the phosphorylation status of *Pf*CPDK1 was significantly decreased by *Pf*PKG inhibition (Supplementary Fig. 2). Similarly, probing with an (in-house) phospho-specific antibody to phosphorylated S64 on *Pf*CDPK1 (Fig. 3e,f) demonstrated that phosphorylation at S64 was reduced by Compound 2 treatment in wild-type parasites (Fig. 3e) but not in *Pf*PKG_T618Q_ mutant parasites (Fig. 3f).

That *Pf*PKG might have a regulatory role in invasion was indicated by the fact that glideosome-associated protein-40 (*Pf*GAP-40: PF3D7_0515700, *Pf*GAP-45: PF3D7_1222700) and myosin A ((*Pf*MyoA: PF3D7-1342600); Fig. 2), all of which are components of a motor protein complex called the glideosome that is essential for apicomplexan parasite invasion^32^,^33^, are *Pf*PKG cellular targets. Further analysis of the 23 *Pf*PKG cellular targets with no designated function (labelled ‘uncharacterized proteins' in Fig. 2), using GeneDB (http://www.genedb.org/Homepage) curation, revealed that six of these proteins had ‘Molecular Function' group assignments (based on an extended similarity group method^34^ in the Gene Ontology data) identical to *Pf*MyoA. The shared Gene Ontology groups were: ‘Motor Activity', ‘Actin Binding' and ‘ATP Binding'. These six proteins also shared the ‘Myosin Complex Cellular Component' group with *Pf*MyoA. These observations may indicate that in addition to *Pf*GAP-40, *Pf*GAP-45 and *Pf*MyoA, other motor-associated proteins could be regulated by *Pf*PKG.

**Figure 4:**
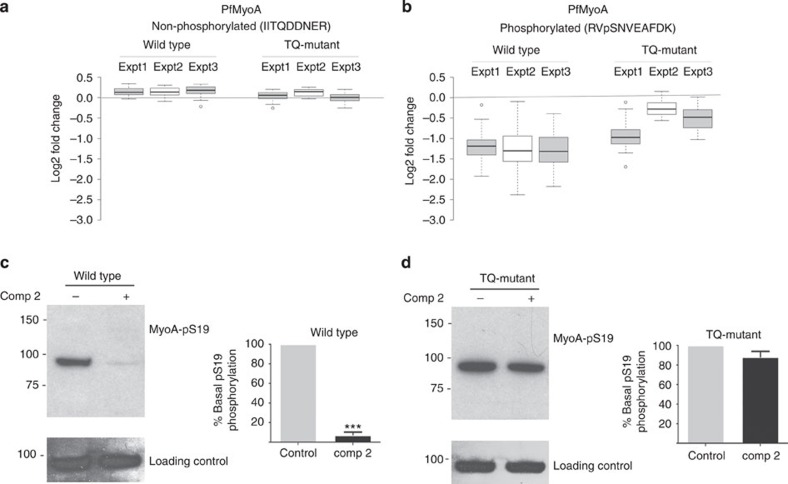
Parasite myosin A (*Pf*MyoA) is a cellular target for *Pf*PKG. (**a,b**) Box plot display of the quantitative changes in abundance of (**a**) a non-phosphorylated *Pf*MyoA peptide derived from wild-type and *Pf*PKG_T618Q_ parasites treated with vehicle or Compound 2 (2 μM; Comp 2) and (**b**) of a peptide containing the serine-19 phosphorylation of *Pf*MyoA. (**c,d**) Western blot of parasite lysates prepared from (**c**) wild-type parasites and (**d**) *Pf*PKG_T618Q_ parasites, treated with vehicle or Comp 2 (2 μM). The blots were probed either with a phospho-specific antibody to phosphorylated serine-19 on *Pf*MyoA (MyoA-pS19) or with an antibody against *Pf*MyoA as a loading control. The western blot data shown are representative of three independent experiments and the quantification of *n*=3 experiments ±s.e.m. Student's paired *t*-test was applied to test statistical significance ***P<0.001.

We further characterized the phosphorylation of *Pf*MyoA at serine 19 (S19), as this phosphorylation event had previously been correlated with calcium^35^ and PKA-dependent phosphorylation^9^ and invasion^35^. Here we confirmed our mass spectrometry data (Fig. 4a,b) by using an in-house phospho-specific antibody to S19 phosphorylation on *Pf*MyoA to monitor the effects of Compound 2 on the phosphorylation of *Pf*MyoA in wild-type and *Pf*PKG_T618Q_ mutant parasites and showing that this phosphorylation event was *Pf*PKG-dependent (Fig. 4c,d).

### *Pf*PKG directly mediates phosphorylation of *Pf*CDPK1 *in vitro*

**Figure 5:**
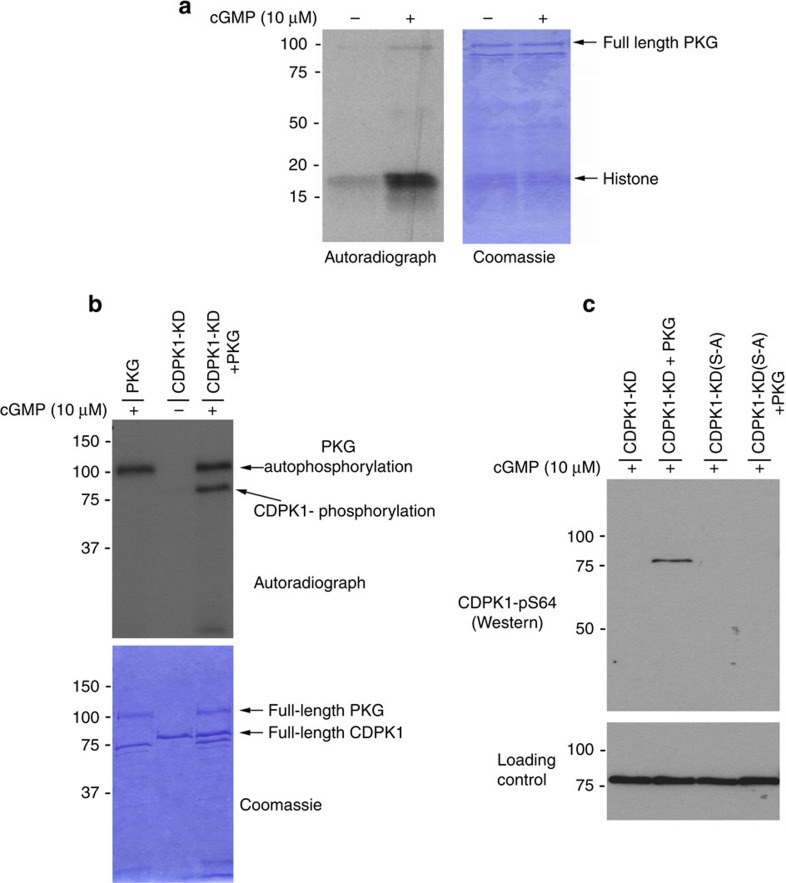
*Pf*CDPK1 is phosphorylated by *Pf*PKG *in vitro*. (**a**) An *in vitro* kinase reaction with [^32^P]-ATP was carried out using a recombinant HIS-tagged *Pf*PKG (1 μg) with histone (2 μg) as a substrate in the presence and absence of cGMP (10 μM). (**b**) An *in vitro* kinase reaction with GST-tagged ‘kinase dead' mutant of *Pf*CDPK1 where asparate-191 was substituted for an asparagine (CDPK1-KD, 1 μg). This construct was used as a substrate for purified recombinant HIS-tagged *Pf*PKG (1 μg) in the presence and absence of cGMP (10 μM). Coomassie-stained gel is shown as a loading control. (**c**) *In vitro Pf*PKG kinase assay performed with recombinant GST-tagged CDPK1-KD (50 ng) as a substrate or a mutant version of CDPK1-KD where serine-64 was substituted by alanine (CDPK1-KD(S-A); 50 ng). The kinase assay was conducted either in the presence or absence of *Pf*PKG (50 ng) and the resulting reaction probed with a phospho-specific antibody to phosphoserine-64 on CDPK1 (CDPK1-pS64). Blots were stripped and probed with a structural *Pf*CDPK1 antibody as a loading control. The results shown are representative of three independent experiments.

The live parasite experiments described above indicate that *Pf*CDPK1 is phosphorylated in a *Pf*PKG-dependent manner, but this may not be due to the direct action of *Pf*PKG on *Pf*CDPK1 but rather might be indirect through the action of another kinase that is itself regulated by *Pf*PKG. To test this we asked if *Pf*PKG could directly phosphorylate *Pf*CDPK1. First, we established that purified recombinant HIS-tagged *Pf*PKG was active and could be stimulated by cGMP (Fig. 5a). We then generated a catalytically inactive mutant of *Pf*CDPK1 by mutating the catalytic asparate 191 to asparagine (Supplementary Fig. 3) and used this kinase dead version (CDPK1-KD) as a substrate for recombinant *Pf*PKG in an *in vitro* kinase assay. These experiments revealed that *Pf*PKG could directly phosphorylate *Pf*CDPK1 *in vitro* (Fig. 5b).

Western blots using the anti-phospho-S64 *Pf*CDPK1 antibody determined that S64 was at least one of the sites on *Pf*CDPK1 phosphorylated by *Pf*PKG (Fig. 5c). Mass spectrometry further confirmed that *Pf*PKG could directly phosphorylate *Pf*CDPK1 at S64 (Supplementary Fig. 4). Interestingly, these experiments also determined that *Pf*PKG could phosphorylate *Pf*CDPK1 at threonine 231 in the activation loop (Supplementary Fig. 4). The biological significance of this second phosphorylation site is, however, unclear since despite extensive efforts we were not able to detect this phosphorylation event *in vivo*.

Serine 64 on *Pf*CDPK1 resides within the glycine-rich loop of domain I, a region crucial for ATP binding. When residues in this loop are phosphorylated in certain mammalian kinases, for example, the cyclin-dependent protein kinase cdk1 (refs 36, 37, 38), this results in abolition of enzymatic activity. We therefore tested whether *Pf*PKG could regulate the activity of *Pf*CDPK1 through phosphorylation of S64. We found that purified recombinant *Pf*CDPK1 was highly active and able to auto-phosphorylate on many residues including S64. Substitution of S64 to an alanine residue (S-A) did not significantly change the enzymatic activity of recombinant *Pf*CDPK1, or the *in vitro* substrate specificity (Supplementary Fig. 5). Furthermore, pre-incubation of *Pf*CDPK1 with *Pf*PKG resulted in a reduction in *Pf*CDPK1 activity, however, the same reduction was also observed in the S-A mutant of *Pf*CDPK1 (Supplementary Fig. 5). Hence, it would appear that *Pf*PKG can decrease the activity of *Pf*CDPK1 *in vitro*, but this does not occur via S64 phosphorylation.

It is possible that the reduction of *Pf*CDPK1 activity in the presence of *Pf*PKG is due to the fact that *Pf*CDPK1 is also phosphorylated by *Pf*PKG at T231 (Supplementary Fig. 4). We attempted to test this by mutating T231 to alanine, however, this *Pf*CDPK1 mutant showed no enzymatic activity. Hence, it would seem that *Pf*PKG can reduce the activity of *Pf*CDPK1 but this is not via S64 phosphorylation but may be via T231 phosphorylation.

### Phosphorylated *Pf*CDPK1 is associated with apical structures

**Figure 6:**
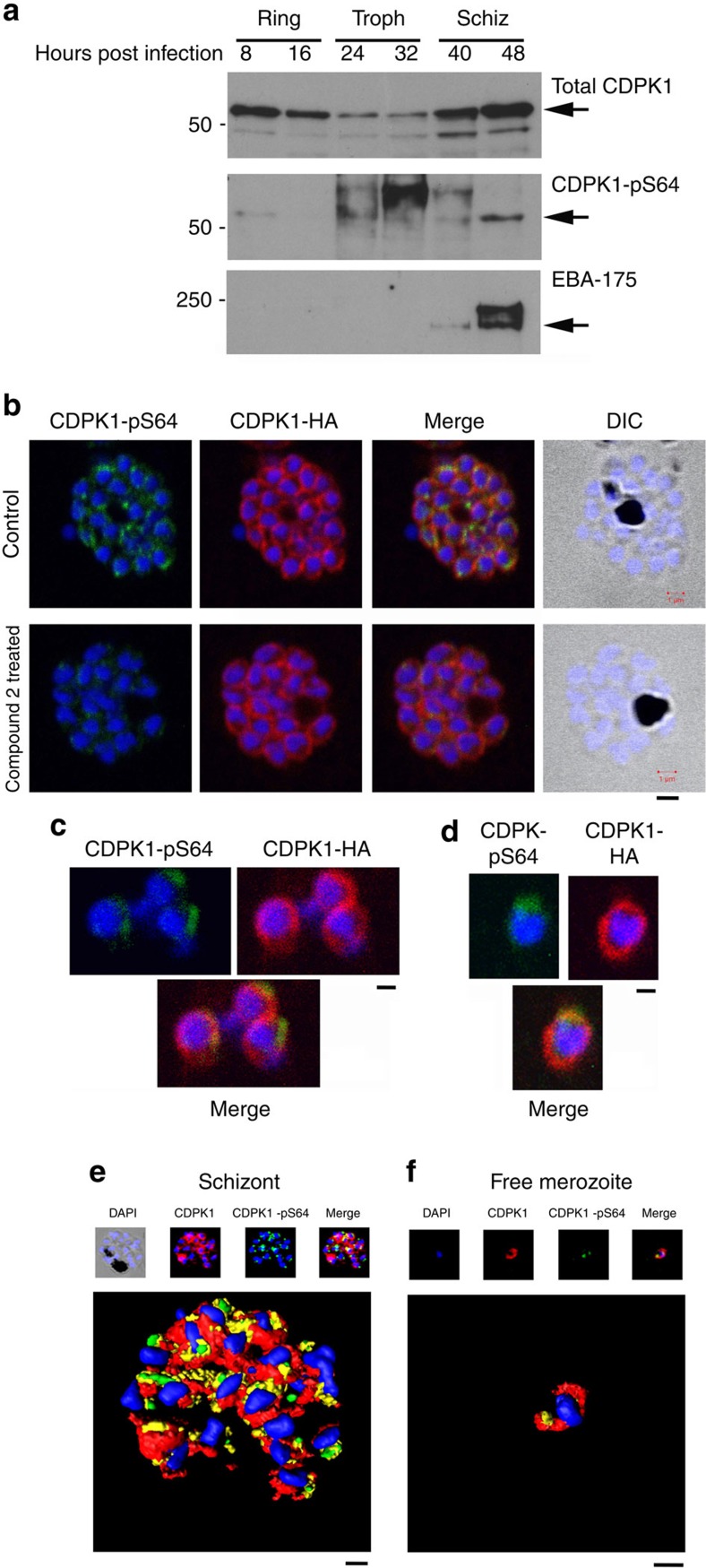
Phosphorylation of *Pf*CDPK1 at S64 occurs during the schizont (Schiz) stage of parasite development. (**a**) Parasite lysates (15 μg) prepared from ring, trophozoite (Troph) or Schiz stage and were probed with the structural *Pf*CDPK1 antibody, the phospho-specific CDPK1-pS64 antibody or the Schiz-specific marker *Pf*EBA-175. (**b**) A late Schiz culture treated with vehicle or Compound 2 (2μM; 60min) was fixed and probed with DAPI stain to reveal the nuclei (blue), the anti-HA antibody to reveal *Pf*CDPK1-HA expressed in transgenic parasites (red) or the phospho-specific CDPK1-pS64 antibody (green). A merge of images from all three stains is shown as well as a differential interference contrast (DIC) image. (**c**,**d**) Free merozoites were fixed and probed with DAPI stain to reveal the nuclei (blue), the anti-HA antibody to reveal *Pf*CDPK1-HA (red) or the phospho-specific CDPK1-pS64 antibody (green). A merge of images from all three stains is shown. (**e**) A rendered image where deconvoluted z stacks (see; Supplementary Movie 1) were reconstructed in 3D, with interpolation, of wild-type parasites stained with DAPI (blue), the structural CDPK1 antibody to reveal the total pool of *Pf*CDPK1 (red) and the CDPK1-pS64 antibody (green). (**f**) Same as **e** but in this case the images (see; Supplementary Movie 2—video for z stacks) are of a free merozoite. These results are representative of at least three experiments. Scale bars, **b**=1 μm, **c**=0.5 μm, **d**=0.5 μm, **e**=1 μm, **f**=0.5 μm

We show here that *Pf*CDPK1 was present in infected RBCs at ring and schizont stage of the erythrocytic asexual cycle as reported previously^39^,^40^ and to a lesser extent in the trophozoite stage (Fig. 6a). This is compared with phosphorylation on S64 that appears to predominantly occur at late schizont stage and be weakly evident at ring stage (Fig. 6a). The synchronization of the parasite culture was confirmed by blotting for the schizont-specific expression of *Pf*EBA-175, which appears in our cultures at 40 h post infection consistent with the parasite culture entering the schizont stage (Fig. 6a).

Immunofluorescence using an anti-HA antibody that detected *Pf*CDPK1-HA in transgenic parasites revealed the peripheral (membrane) localization of *Pf*CDPK1 in the nascent merozoites contained within the late-stage schizonts or free merozoites, in agreement with previous studies^39^,^40^ (Fig. 6b). This localization was evident in transgenic parasites expressing *Pf*CDPK1-HA, and stained with anti-HA antibody (Fig. 6b–d), as well as in wild-type parasites stained with our structural CDPK1 antibody (Fig. 6e,f). In contrast, S64-phosphorylated *Pf*CDPK1 appeared as discrete ‘punctate spots' at the periphery of merozoites within late schizonts (Fig. 6b,e) and within free merozoites (Fig. 6c,d,f). Although it appears that a small proportion of the phosphorylated *Pf*CDPK1 signal does not overlap with the total pool of *Pf*CDPK1, and this may represent a level of nonspecific background, immunostaining for CDPK1-pS64 was substantially reduced following 1 h treatment with Compound 2 (Fig. 6b), confirming the specificity of the pS64 phospho-specific antibody.

**Figure 7:**
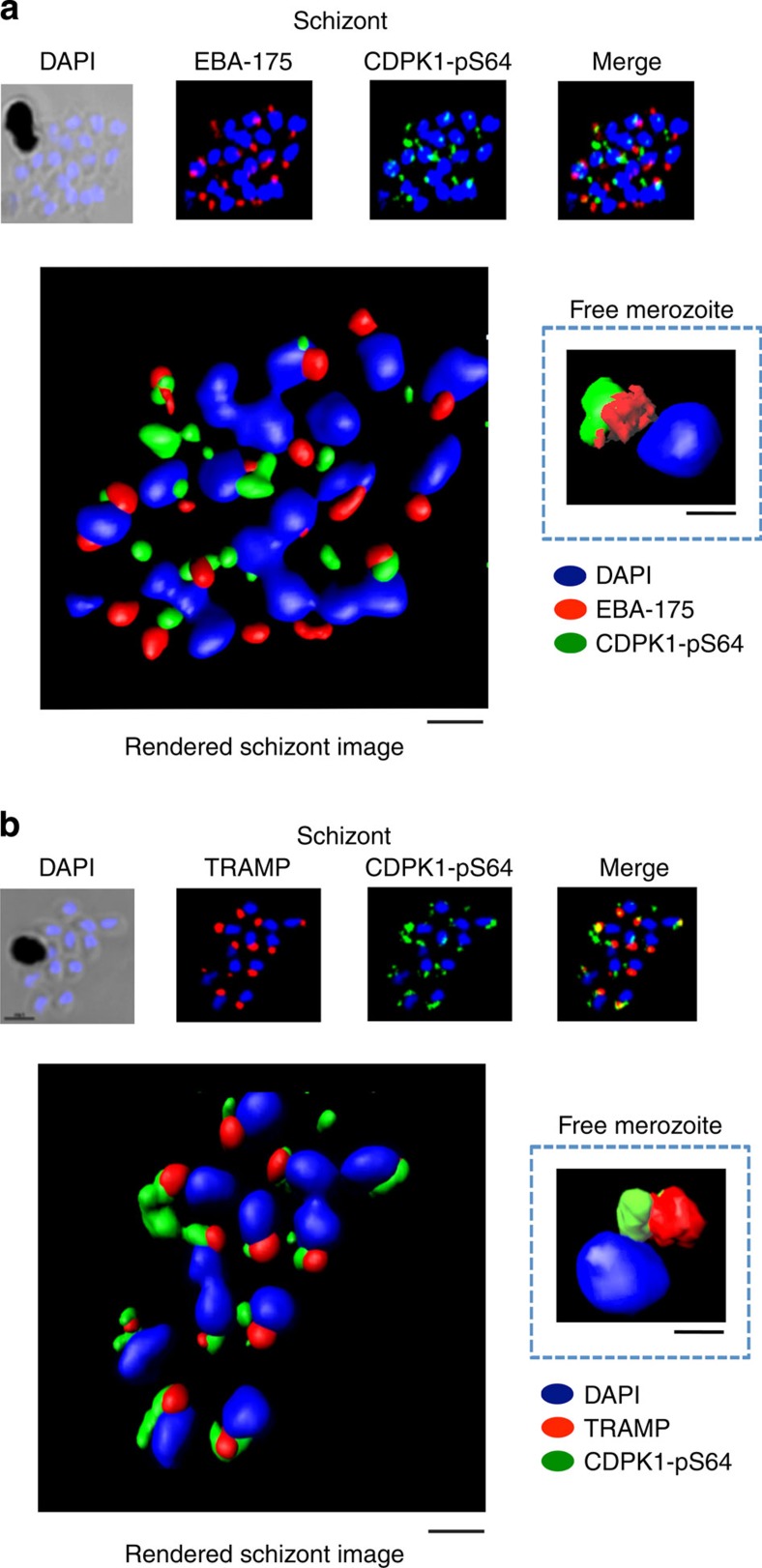
*Pf*CDPK1 phosphorylated at serine-64 is associated with apical parasite structures. (**a**) A schizont stage parasite stained with DAPI to reveal the nuclei (blue), an antibody to *Pf*EBA-175 to reveal the micronemes (red) and the phospho-specific CDPK1-pS64 antibody (green). A merge of images from all three stains is shown on the far right; this is a representative image from at least three experiments. Also shown is a rendered image where deconvoluted z stacks (see: Supplementary Movie 3) were reconstructed in 3D, with interpolation. The inset shows a rendered image of a free merozoite from the same preparation (see: Supplementary Movie 4). (**b**) The same as **a**, but instead of probing with an anti-EBA-175 antibody the preparation was probed with an antibody to the rhoptry marker *Pf*TRAMP (red;see: Supplementary Movie 5 (schizonts) and Supplementary Movie 6 (merozoites) for z stacks). Scale bars, **a**=1 μm (insert=0.5 μm), **b**=1 μm (insert=0.5 μm),

Furthermore, the phosphorylated form of *Pf*CDPK1 in merozoites contained within late-stage schizonts or free merozoites was localized close to the micronemes (labelled with anti-EBA-175 antibodies^41^) and rhoptries (labelled with anti-*Pf*TRAMP antibodies^42^). It is important to note that the phosphorylated *Pf*CDPK1 does not appear within the apical organelle structures themselves (correlation coefficient for EBA175 and CDPK1-pS64 in merozoites=0.301 and for TRAMP and CDPK1-pS64=0.414) but rather is located close to these structures. Thus, we conclude that the phosphorylated form of *Pf*CDPK1 is largely located at the apical pole of the parasite (Fig. 7a,b). (Z-stacks for the 3D-reconstructed figures presented in Fig. 6e,f as well as in Fig. 7a,b are shown in Supplementary Movies 1–6.)

### Phosphorylated *Pf*CDPK1 contributes to complex formation

**Figure 8:**
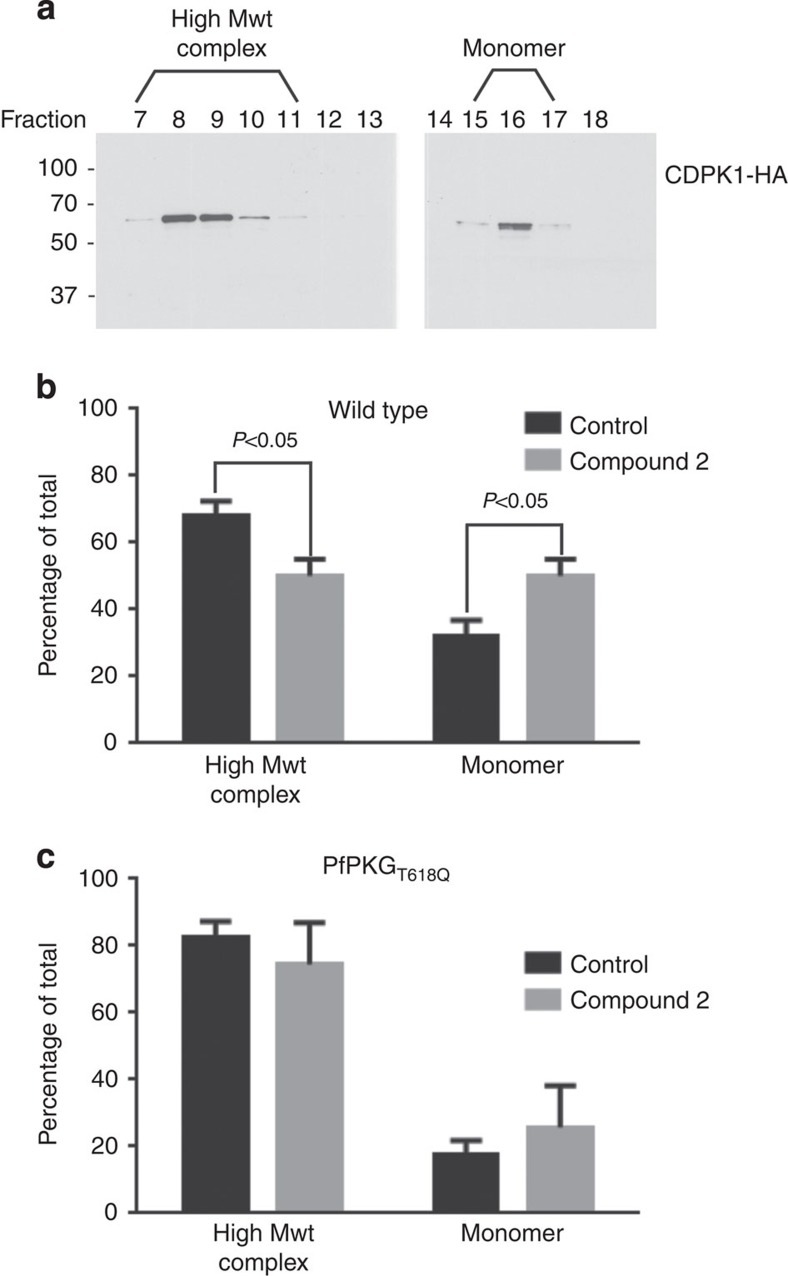
*Pf*CDPK1 exists as a high-molecular-weight (Mwt) complex that is regulated by *Pf*PKG. (**a**) A parasite lysate was prepared from parasites expressing a HA-tagged *Pf*CDPK1. The lysate was fractionated over a Superdex 200 gel filtration column and the fractions probed by western blot with an anti-HA antibody. Shown is a representative of three independent experiments. (**b**) Quantification of the relative proportion of CDPK1-HA staining that appears in the high-Mwt complex and in the monomeric form—from parasites treated with vehicle and from parasites treated with Compound 2. (**c**) The experiment shown in **b** was repeated using the *Pf*PKG_T618Q_ mutant parasites. In this instance, the fractions from the Superdex column were probed with the structural CDPK1 antibody to detect the high-Mwt complex and the monomeric form of *Pf*CDPK1. The results shown are from three experiments ±s.e.m. Student's paired *t*-test was applied to test statistical significance.

The possibility that phosphorylation of *Pf*CDPK1 might regulate protein–protein interactions was tested by size exclusion chromatography where *Pf*CDPK1 was observed to exist as both a ∼60-kDa monomer and a high-molecular-weight complex running between 250 and 400 kDa (Fig. 8a). Treatment of parasites with Compound 2 appeared to have a subtle but significant effect on complex formation as indicated by a reduction in the proportion of *Pf*CDPK1 in the high-molecular-weight complex and an increase in the proportion of monomeric *Pf*CDPK1 (Fig. 8b). This reduction in response to Compound 2 was not evident in *Pf*PKG_T618Q_ mutant parasites (Fig. 8c) indicating that S64 phosphorylation mediated by *Pf*PKG is involved in maintaining a proportion of *Pf*CDPK1 within a high-molecular-weight complex, although the precise nature of the complex has still to be defined.

It was decided to probe the possibility that *Pf*PKG could be part of the high-molecular-weight *Pf*CDPK1 complex. However, probing the gel filtration fractions for *Pf*PKG revealed that the kinase was only present in the low-molecular-weight fractions consistent with *Pf*PKG monomer and therefore was not part of any high–molecular-weight complex with *Pf*CDPK1 (Supplementary Fig. 6).

### *Pf*PKG activity is essential for merozoites RBC invasion

**Figure 9:**
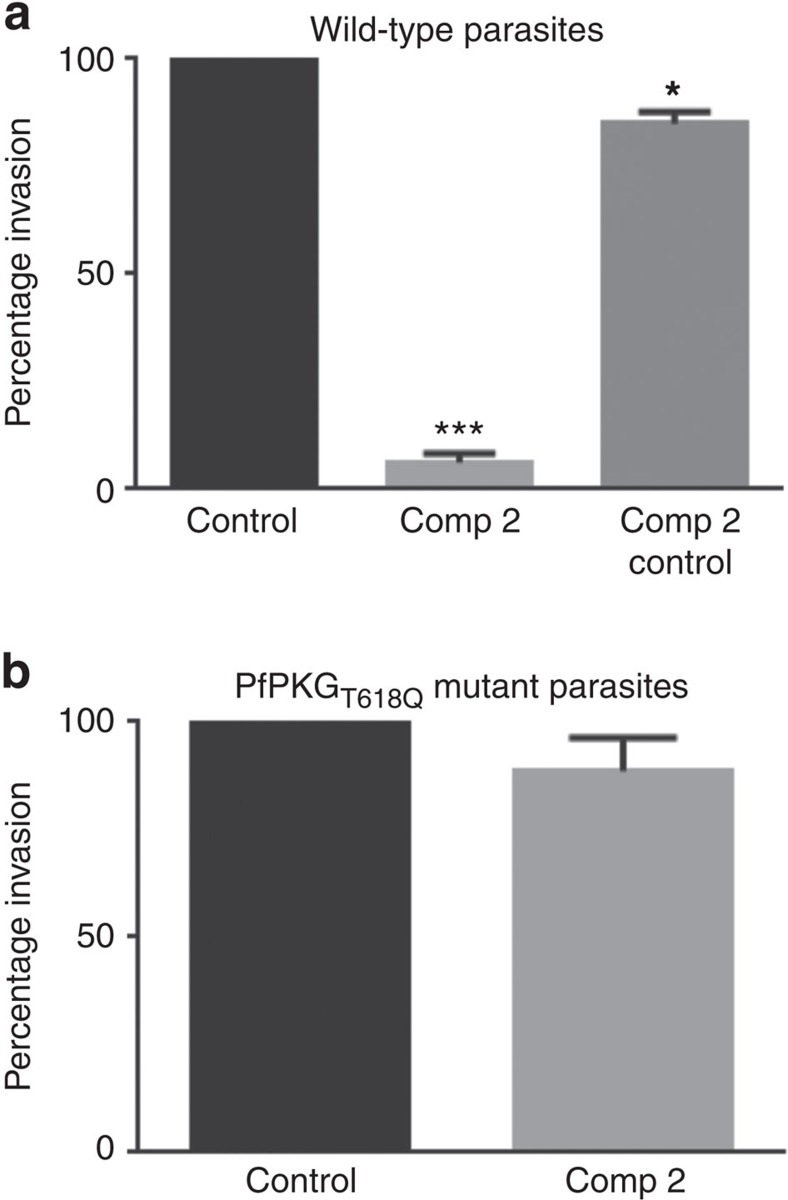
Parasite red blood cell invasion is dependent on *Pf*PKG activity. (**a**) Invasion of wild-type free merozoites into red blood cells was monitored in the absence or presence of Compound 2 (2 μM; Comp 2) by the determination of the number of infected red blood cells 24 h post invasion. Comp 2 was also added 1 h post infection and the number of infected red blood cells 24 h later was determined (Comp 2 Control); this was to control for the possibility that Comp 2 treatment might affect the survival of the parasites post invasion. (**b**) The invasion of *Pf*PKG_T618Q_ merozoites into red blood cells was monitored. The results are the mean of 3–5 experiments ±s.e.m. Student's paired *t*-test was applied to test statistical significance. ***P<0.001, *P<0.05.

We determine here that a number of *Pf*PKG cellular targets, not least *Pf*CDPK1 and components of the glideosome complex, are crucially involved in parasite invasion. These data support the notion that *Pf*PKG regulates invasion. To test this, we conducted an invasion assay that monitored the invasion of free merozoites (separated from schizonts by 1.2 μm pore filtration) into RBCs (Fig. 9). In this experiment, Compound 2 completely inhibited invasion of wild-type merozoites but had only a small effect on invasion of *Pf*PKG_T618Q_ mutants merozoites (Fig. 9a,b). These data are consistent with a role for *Pf*PKG in invasion and supports the notion that the phosphorylation of key proteins involved in the invasion process such as *Pf*MyoA and *Pf*CDPK1 might contribute to the mechanism of action of *Pf*PKG.

## Discussion

By employing a chemical genetic approach in conjunction with quantitative global phosphoproteomics, we describe here 107 phosphorylation events in the human malaria parasite that are dependent on *Pf*PKG activity. In this way, we not only reveal the cellular targets (either direct or indirect) of cGMP-signalling in malaria, but we also provide a map of the physiological processes that are likely regulated by *Pf*PKG (see, Fig. 2). In addition, by defining cellular effectors of *Pf*PKG we have gained insight into cGMP/*Pf*PKG phospho-signalling pathways and feed-back loops, and particularly the relationship between *Pf*PKG signalling and calcium signalling in malaria parasites.

**Figure 10:**
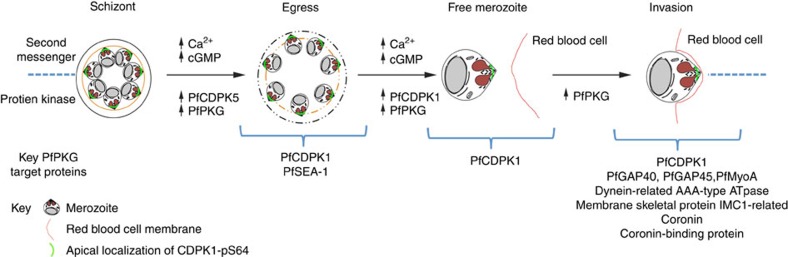
Schematic representation of the role of cGMP/*Pf*PKG signalling in egress/invasion. The present study has identified a number of cellular targets for *Pf*PKG, which appear to map to the reported roles for cGMP and calcium signalling in egress and invasion. We describe here the *Pf*PKG-dependent phosphorylation of a subpopulation of *Pf*CDPK1 localized at the apical pole, which together with other calcium-dependent kinases, most notably *Pf*CDPK5, represents an interplay between calcium and cGMP/*Pf*PKG signalling necessary to mediate egress. Interestingly, this may also involve phosphorylation of additional proteins involved in egress, such as the recently described *Pf*SEA1, which we demonstrate here to be a target (either direct or indirect) for *Pf*PKG. Furthermore, the identification of a number of proteins involved in parasite invasion, particularly those associated with the actomyosin motor complex, provides a mechanistic explanation for the essential role played by *Pf*PKG in invasion that is described in our study.

This relationship was particularly evident in parasite egress and invasion. Previous studies had demonstrated that intracellular calcium levels rise sharply before parasite egress^43^, a process that is blocked by chelating intracellular calcium with BAPTA-AM^15^,^43^. The rise in calcium has been proposed to initiate the release of exoneme proteins, in particular, the subtilisin-like serine protease *Pf*SUB1 (ref. 15) and microneme proteins (for example, *Pf*AMA1 and *Pf*EBA175)^44^, in a process that is essential for egress and invasion^15^,^44^,^45^. That this also involves *Pf*PKG is evident by our previous report that increasing parasite intracellular concentrations of cGMP, by inhibition of parasite cGMP-phosphodiesterase with zaprinast, resulted in parasite egress in a *Pf*PKG-dependent manner^15^. Furthermore, microneme discharge and parasite egress can be prevented by the treatment of parasites with Compound 2 (ref. 15) and by inhibition of *Pf*CDPK1 (refs 46, 47). It would seem, therefore, that there is a close relationship between changes in intracellular calcium, calcium signalling through *Pf*CDPK1 and the activity of *Pf*PKG, in the regulation of exoneme and microneme discharge associated with egress and invasion. Our studies would indicate that one important component of this relationship is the ability of *Pf*PKG to phosphorylate a sub-population of *Pf*CDPK1 at the apical pole of the parasite. These data, together with fact that *Pf*PKG-dependent phosphorylation of *Pf*CDPK1 impacts on the nature of the *Pf*CDPK1-complex, present the possibility *Pf*PKG exerts regulation of microneme release and parasite invasion by the phosphorylation of *Pf*CDPK1 at the apical pole (Fig. 10).

Such a mechanism may well run in conjunction with other processes orchestrated by *Pf*PKG. In particular, recent studies in both *P. berghei* and *P. falciparum* have determined that *Pf*PKG can mediate changes in intracellular calcium via the regulation of phosphoinositide biosynthesis^18^—the lipid precursors of inositol trisphosphate (IP_3_) that is proposed to act in *Plasmodium* in a similar way to mammalian cells, to mobilize intracellular calcium^44^,^48^. It is therefore possible that increased *Pf*PKG activity before egress can initiate not only the *Pf*CDPK1 phosphorylation and complex formation but also propagate an increase in intracellular calcium that activates calcium-dependent phosphorylation, which subsequently contributes to microneme discharge and parasite egress. Thus, our study, in conjunction with others, highlights the complex relationship between cGMP/*Pf*PKG and calcium/*Pf*CDPK1 signalling in the regulation of parasite egress and provides direct evidence for the involvement of phospho-signalling cascades in this key process.

The possibility that *Pf*PKG can impact further on parasite egress is evident by our intriguing finding that schizont egress antigen-1 (*Pf*SEA-1, PF3D7_1021800) is phosphorylated in a *Pf*PKG-dependent manner. Antibodies to *Pf*SEA-1 have recently been reported in Tanzanian children resistant to severe malaria^49^, where a block of *Pf*SEA-1 has been proposed to inhibit merozoite egress^49^. We identified here 11 sites of phosphorylation on *Pf*SEA-1, of which serine-280 in the N-terminal region was phosphorylated in a *Pf*PKG-dependent manner—providing an additional potential mechanism for *Pf*PKG-mediated regulation of parasite egress.

That *Pf*PKG-mediated signalling has an impact on invasion is evidenced here by the *Pf*PKG-mediated phosphorylation of *Pf*MyoA, *Pf*GAP40 and *Pf*GAP45, crucial components of the glideosome complex^40^. In particular, *Pf*GAP45 was found to be a substrate for *Pf*CDPK1, which appears to phosphorylate *Pf*GAP45 at serine-89 and serine-103 (ref. 50). Interestingly, we identified both sites as phosphorylated in our *in vivo* study, but they were not significantly changed by treating parasites with Compound 2 and therefore were not *Pf*PKG-dependent. Surprisingly, we identified two additional sites serine-149 and serine-156 that were *Pf*PKG-dependent, indicating that calcium/*Pf*CDPK1 signalling and cGMP/*Pf*PKG signalling in this instance converge in the regulation of *Pf*GAP45 by acting at independent sites. The relationship between cGMP and calcium signalling in the regulation of *Pf*GAP45 may extend further. It has been reported that *Pf*GAP45 can be phosphorylated by *Pf*PKB, which, unlike the mammalian orthologue that acts downstream of phosphoinositides, is regulated in a calcium/calmodulin-dependent manner^51^. Of further interest was the phosphorylation of *Pf*MyoA at S19, a site identified as phosphorylated *in vivo* in all the previous global phosphoproteomic studies^9^,^10^,^11^,^12^ and suggested to be a *Pf*PKA site based on (i) the close match to the consensus PKA sequence and (ii) the observation that a peptide corresponding to this site was phosphorylated by mammalian PKA^9^. Our studies would indicate that this site is phosphorylated in a *Pf*PKG-dependent manner, although it is not possible to determine at this stage if this is a direct action of *Pf*PKG or whether *Pf*PKG upregulates the activity of another kinase, such as *Pf*PKA, and that this kinase then phosphorylates *Pf*MyoA. This might be possible as our studies demonstrate that one of the adenylyl cyclases (PfACβ) and a phosphodiesterase (PDEβ) were phosphorylated in a *Pf*PKG-dependent manner, which may provide a mechanism through which *Pf*PKG could impact on cAMP/*Pf*PKA signalling.

Thus, our results suggest that *Pf*PKG regulates parasite invasion, a notion that was upheld in our study when Compound 2 was shown to inhibit invasion of free merozoites into RBCs. Importantly, the effect of Compound 2 was clearly mediated by *Pf*PKG, as its effect on parasite invasion was significantly reduced in the *Pf*PKG_T618Q_ mutant parasite strain. Hence, we show here that *Pf*PKG activity is essential for parasite invasion and that this correlates with the identification of a number of key proteins involved in parasite invasion that are phosphorylated in a *Pf*PKG-dependent manner (Fig. 10).

In conclusion, our study is among the first to use quantitative phospho-proteomics in combination with chemical genetics to dissect phospho-signalling in malaria and extends significantly our understanding of the role played by phosphorylation from the previously published global phospho-proteomic studies that simply identified parasite phospho-proteins^9^,^10^,^11^,^12^,^52^. By employing the techniques described here, we establish that malaria protein kinases can reside within phospho-signalling cascades and, similar to mammalian systems, a certain number of these parasite kinases form signalling hubs able to interact with a large number of downstream systems. It would appear that *Pf*PKG represents one of these essential signalling hubs where signalling through cyclic nucleotides, calcium and phosphoinositides^16^,^18^ appear to converge. In this context it would seem that targeting *Pf*PKG would have a wide impact on essential parasite signalling. That this is the case is supported by the fact that *Pf*PKG is an essential kinase^11^,^14^ and that inhibition of this kinase is shown here to prevent merozoite invasion in addition to other key life cycle events such as schizogony^14^, egress^15^,^16^, gametogenesis^17^ and ookinete motility^18^,^19^. These widespread effects of inhibition of *Pf*PKG activity supports our notion that *Pf*PKG acts as an essential signalling hub and as such would represent an excellent drug target in the treatment of malaria and blocking its transmission to mosquitoes.

## Methods

### Parasite culture and synchronization of parasites

*P. falciparum* blood stage 3D7 (wild type)-, PKG_T618Q_- and CDPK1-HA-parasites were cultured using a standard method^53^. Parasites were grown in complete RPMI 1640 medium (RPMI 1640 medium with 2 mM L-glutamine, 25 mM HEPES, 2 g l^−1^ NaHCO_3_, 27.2 mg l^−1^ hypoxanthine and 0.5% Albumax II, pH7.4) using O^+^ human RBC at 37 °C in an incubator with 5% CO_2_, 5% O_2_ and 90% N_2_. PKG_T618Q_ and CDPK1-HA parasites were grown with the selection drug WR99210 (10 nM). Sorbitol treatment was used to synchronize the parasites^54^: parasites were treated with 5% sorbitol for 20 min at room temperature to lyse trophozoite and schizont stage parasites. Dead parasites were removed by two washes with incomplete RPMI medium (RPMI 1640 medium with 2 mM L-glutamine, 25 mM HEPES, pH 7.4). Following sorbitol treatment parasites were transferred to complete RPMI 1640 medium.

For the time-course experiments, parasites were synchronized by two rounds of sorbitol treatment—first treatment when the parasites culture was at late ring/trophozoite stage and second when the parasite culture contained schizonts and ring stage parasites. After second sorbitol treatment, parasite cultures were collected for the first time point (8 h) and further samples were collected at every 8 h as indicated. Please note that we calculated that each time point has variation of ±2 h. Parasites from infected cells for the first three time points (8, 16 and 24 h) were collected by two saponin treatments (0.1%) for 10 min. Subsequent time points (32, 40 and 48 h) were collected by magnet-assisted cell sorter (MACS) purification followed by saponin treatment (0.1%) for 10 min. The parasite fractions were then washed at least three times with PBS before being prepared for gel electrophoresis.

### Cloning of CDPK1 and site-directed mutagenesis

Bacterial expression of full-length *Pf*CDPK1 was achieved by amplification of the *Pfcdpk1*-coding sequence from a 3D7 schizonts cDNA preparation using CDPK1-FL-His-Fwd and CDPK1-FL-His-Rev primers (see primer list) and cloning into pLEICS05 plasmid (PROTEX, University of Leicester, UK), which produces a *Pf*CDPK1 protein with a 6-HIS tag at the C-terminus. For expression of *Pf*CDPK1 as an N-terminal GST-tagged protein, the *Pfcdpk1* gene was amplified using CDPK1-FL-GST-Fwd and CDPK1-FL-GST-Rev primers and cloned in pGEX-2T plasmid (GE Healthcare). Site-directed mutagenesis (QuikChange II kit, Agilent Technologies), using primers CDPK1-D191N-Fwd and CDPK1-D191N-Rev, was performed to convert aspartate to asparagine at residue-191 of *Pf*CDPK1 to generate the kinase dead variant, CDPK1-KD. Similarly, serine-64 on *Pf*CDPK1 was changed to alanine using the primers CDPK1-S64A-Fwd and CDPK1-S64A-Rev primers.

### Generation and screening of parasites expressing CDPK1-HA

A plasmid containing a recodonized version of base pairs 436–1,572 of the *pfcdpk1* ORF between XmaI (5′) and AvrII (3′) cloning sites was synthesized by GeneArt. The synthetic sequence also contained a novel *Eco*RI site at position 436–441, produced through introduction of synonymous mutations. A region of homology to facilitate integration of the plasmid via single crossover homologous recombination was amplified from *P. falciparum* 3D7 genomic DNA: 194 bp upstream of the ATG to base pair 435 of the open reading frame using primers 1 and 2. This fragment was cloned via XmaI and EcoRI sites into the GeneArt vector containing the recodonized gene. Finally, the combined 2.5 kb fragment containing the native and recodonized *cdpk1* sequences was cloned between XmaI and AvrII sites of the pHH4-HA plasmid (gift from Dr E. Knuepfer, NIMR), which adds a triple HA tag at the 3′ end followed by a stop codon and the PbDT 3′ UTR and hDHFR drug selection cassette.

*P. falciparum* 3D7 parasites were transfected with 100 μg pHH4-CDPK1-HA plasmid using standard methods^55^,^56^. They were maintained under drug pressure (25 nM WR99210) until resistant parasites emerged, cycled on/off drug and cloned by limiting dilution. Clones were screened by PCR using primers 4 and 5 to detect integration at the *cdpk1* locus, and primers 4 and 6 which would amplify from the unmodified locus (Supplementary Fig. 7). Positive clones were confirmed by western blot using anti-CDPK1 (described elsewhere^40^ and anti-HA (3F10; Roche) antibodies.

### Immunofluorescence assays

Smears were prepared from *Pf*CDPK1-HA transgenic parasite culture containing schizonts after treatment with vehicle or Compound 2 (2 μM, 60 min) and 3D7 parasite culture containing schizonts and merozoites. Smears were dried for 30 min and washed with Tris-buffered saline (TBS) for 10 min. Smears were fixed with 4% paraformaldehyde (PFA) for 10 min, washed with TBS and permeabilized with 0.1% Triton X-100. Slides were blocked overnight at 4 °C using 4% BSA. Smears were probed with a rabbit antisera raised against phosphoserine-64 on *Pf*CDPK1 (CDPK1-pS64), a monoclonal rat anti-HA antibody (clone 3F10) and a rat antibody against recombinant *Pf*CDPK1 (CDPK1) at 1:100 dilution in TBS containing 1% BSA. Mouse sera against *Pf*EBA175 and *Pf*TRAMP were also used at 1:100 dilution in TBS containing 1% BSA as markers for apical organelles. Alexa-Fluor 546-conjugated anti-rat IgG goat antibodies (1:1,000 dilution) and Alexa-Fluor 488-conjugated anti-rabbit IgG goat antibodies (1:1,000 dilution) were used as secondary antibodies with *Pf*CDPK1-HA transgenic parasite. Alexa-Fluor 594-conjugated anti-rat IgG goat antibodies (1:500 dilution), Alexa-Fluor 594-conjugated anti-mouse IgG goat antibodies (1:500 dilution) and Alexa-Fluor 488-conjugated anti-rabbit IgG goat antibodies (1:200 dilution) were used as secondary antibodies with 3D7 parasites. Slides were mounted with 4′,6-diamidino-2-phenylindole dihydrochloride (DAPI) and anti-fade mounting medium (Molecular Probes) and analysed using either a Nikon A1-R confocal microscope (for 3D7 parasites) or a Zeiss Axio Observer Z1 confocal microscope (for *Pf*CDPK1-HA parasites).

Images (labelled as rendered images in the manuscript) were processed using Imaris version 6.4.2 or 7.0.0 (Bitplane Scientific), which enables visualization, segmentation and interpretation of three-dimensional (3D) microscopy data sets. For clarity of display, deconvoluted z stacks were reconstructed in 3D, with interpolation. To see the extent of co-localization between *Pf*CDPK1-pS64 (green) and *Pf*CDPK1 (red) a separate co-localization channel (shown in yellow; *Pf*CDPK1+*Pf*CDPK1-pS64) was built. The red channel (for *Pf*CDPK1) was made transparent to allow visualization of DAPI and staining for *Pf*CDPK1 (green) or overlap of *Pf*CDPK1-pS64 and *Pf*CDPK1.

### Bacterial protein expression

Full-length *Pf*CDPK1 as a 6-HIS- or GST-tagged protein was expressed in the BL21-CodonPlus(DE3)-RIPL strain (Agilent Technologies) of *E. coli*. Cells were treated with 100 μM IPTG when the optical density of the cell culture at 600 nm reached 0.6, and gene expression was induced for 4 h at 22 °C.

To purify 6-HIS-tagged CDPK1, cells were lysed in buffer (20 mM Tris-HCl, 150 mM NaCl, 1 mM DTT, Protease Inhibitor Cocktail (Roche) pH7.4) by sonication. After sonication lysates were centrifuged at 20,000*g* for 30 min and supernatants were collected. Supernatants were loaded on pre-equilibrated Ni-NTA Superflow resin (QIAGEN). Loaded resin was washed with buffer (20 mM Tris-HCl, 150 mM NaCl and 1 mM dithiothreitol (DTT), pH 7.4) and proteins eluted with the same buffer containing 350 mM Imidazole, at pH 8.0. Eluted proteins were dialysed against 20 mM Tris-HCl, 150 mM NaCl and 1 mM DTT, pH7.4.

To purify GST-tagged *Pf*CDPK1, cells were lysed in buffer (PBS containing 1 mM DTT, 1 mM EDTA and Protease Inhibitor Cocktail (Roche), pH7.4) by sonication. After sonication, the lysate was centrifuged at 20,000*g* for 30 min and the supernatant collected. Supernatant was loaded on pre-equilibrated glutathione Sepharose-4B resin (GE Healthcare). Loaded resin was washed with PBS containing 1 mM DTT and 1 mM EDTA, pH7.4, and protein was eluted with the same buffer containing 20 mM reduced glutathione. Eluted proteins were dialysed in dialysis buffer and stored at −80 °C.

### Antibody generation

*Pf*CDPK1 ‘structural' antibody (recognizing both phosphorylated and non-phosphorylated *Pf*CDPK1) was raised by immunizing rats with recombinant CDPK1-6HIS protein by Eurogentec, using a standard 87-day immunization protocol. Briefly two rats were immunized with 30 μg of protein on day 0 followed by boosts on days 14, 28 and 56. The final bleed was collected on day 87. IgG from the serum was purified on Protein G-Sepharose fast flow (GE Healthcare) and used for the study.

Phospho-specific Serine-64 *Pf*CDPK1 antibody was raised by immunizing rabbits with phospho-peptide CKVRKLGS(PO_3_H_2_)GAYGE. Rabbit immunizations and purification of phospho-specific antibody was done by Eurogentec following their 87-day immunization protocol. Briefly two rabbits were immunized with peptide coupled to KLH carrier on day 0 followed by three boosts on day 21, 49 and 77, and the final bleed was collected on day 87. ELISA against peptide and carrier was used to select the final serum sample with the best titre for the purification of phospho-specific antibody. Specific antibody was purified by two rounds of purification: first, the serum was passed through a column with conjugated phospho-peptide, then the bound antibodies were eluted and passed through a second column with conjugated non-phosphorylated peptide (CKVRKLGSGAYGE). The flow-through that contained the phospho-specific antibody, as confirmed by ELISA, was collected.

The phosopho-specific antibody to *Pf*MyoA phospho-serine 19 was produced by Genosphere Biotechnologies. Rabbits were immunized with the peptide ‘N'-RRV[pS]NVEAFDKC conjugated to KLH. The resulting antisera was affinity-purified on the phosphopeptide followed by affinity chromatography on the non-phosphopeptide. The flow-through from the second purification contains a majority of anti-phospho-peptide antibodies and was used in western blots at a dilution of 1:1,000.

### Labelling of *Pf*CDPK1-HA parasites and immunoprecipitation

Late schizont-stage *Pf*CDPK1-HA parasites were harvested on a MACS column (Miltenyi Biotec) in Krebs buffer (10 mM HEPES, containing 118.4 mM NaCl, 4.69 mM KCl, 4.17 mM NaHCO_3_, 1.18 mM MgSO_4_, 1.29 mM CaCl_2_ and 11.7 mM glucose, pH7.4). Purified schizonts were pre-incubated with vehicle (0.2% dimethyl sulphoxide) or 2 μM Compound 2 for 10 min at 37 °C, then ^32^P-orthophosphate (7.4 MBq) was added and the incubation continued at 37 °C for 30 min. The schizonts were washed twice with Krebs buffer and resuspended in 0.1% (w/v) saponin in PBS for 10 min on ice. Following saponin treatment, the cells were washed twice with PBS and then lysed in lysis buffer-A (20 mM Tris, 1 mM EDTA, 20 mM β-Glycerol Phosphate, 1% (v/v) Nonidet P-40 and protease and phosphatase inhibitor cocktail (Roche), pH7.4). The lysate was centrifuged at 20,000*g* for 5 min and the supernatant used for immunoprecipitation. To immunoprecipitate CDPK1-HA, the supernatant was incubated with 20 μl HA-affinity matrix (immobilized anti-HA high-affinity rat monoclonal antibody, clone 3F10 (Roche)) at 4 °C for 1 h with constant mixing. The matrix was then washed five times with lysis buffer-A without NP-40, and an equal volume of Laemmli buffer was added to the matrix. Proteins were separated by SDS–polyacrylamide gel electrophoresis (PAGE) on a 10% polyacrylamide gel, and stained with Coomassie blue before the gel was dried and exposed to X-ray film.

### *In vitro* kinase assays for *Pf*PKG

CDPK1-HIS (1 μg) or CDPK1-S64A-HIS (1 μg) proteins were incubated with or without Histone Type IIA (2 μg), Myelin Basic Protein (2 μg; MBP) and α-Casein (2 μg) in kinase buffer (10 mM HEPES, 10 mM MgCl_2_ and 1 mM DTT, pH7.4) containing 1 mM CaCl_2_, 50 μM ATP and 0.0185 MBq ^32^P-ATP for 30 min at 30 °C.

To examine the phosphorylation of *Pf*CDPK1-KD by *Pf*PKG in an *in vitro* kinase assay, recombinant HIS-tagged *Pf*PKG protein (1 μg) was incubated with or without CDPK1-KD protein (1 μg) in kinase buffer containing 10 μM cGMP, 50 μM ATP and 0.0185 MBq P^32^-ATP for 30 min at 37 °C. CDPK1-KD (1 μg) was incubated in a similar condition without cGMP.

Following incubation, reactions were stopped by adding an equal volume of 2 × Laemmli buffer and proteins were separated by SDS–PAGE on a 10% gel and stained with Coomassie blue. Dried gels were exposed to X-ray film and autoradiograms collected.

To examine phosphorylation of *Pf*CDPK1 at serine-64, *Pf*PKG-HIS tagged (50 ng) was incubated with or without CDPK1-KD (50 ng) and CDPK1-S64A-KD (50 ng) in kinase buffer containing 10 μM cGMP and 50 μM ATP for 30 min at 37 °C. CDPK1-KD (50 ng) and CDPK1-S64A-KD (50 ng) were also incubated in a similar condition without cGMP. Following incubation, the reaction was stopped with Laemmli buffer and samples were used for SDS–PAGE and western blotting.

### *Pf*CDPK1 protein kinase activity assay

Recombinant His-tagged WT or S64A variant *Pf*CDPK1 were assayed for protein kinase activity using MBP as a substrate. In the first part of the assay, recombinant *Pf*CDPK1-6His was incubated in the presence or absence of *Pf*PKG-His plus 10 μM cGMP in the reaction mix containing 50 μM ATP and ^32^P-ATP in kinase buffer (20 mM HEPES, 1 mM MgCl_2_, 20 mM β-glycerol phosphate, pH 7.5) for 30 min at 37 °C. Following the incubation, *Pf*PKG-dependent phosphorylation of *Pf*CDPK1 was stopped by adding Compound 2 to a final concentration of 2 μM and further incubated for 10 min at room temperature. The same amount of Compound 2 was added to control samples not containing *Pf*PKG to exclude any inhibitory effect of the compound on *Pf*CDPK1 in the second part of the assay. Protein kinase activity of *Pf*CDPK1 was assayed by transferring 75% of the first reaction mix into a clean Eppendorf tube containing 2 μg MBP, 1 mM CaCl_2_, 50 μM ATP and ^32^P-ATP in the kinase buffer at 30 °C, and incubation for different times. The reaction was stopped by transferring samples into ice and spotting 20 μl aliquots onto P-81 phospho-cellulose paper (Whatman). The P-81 paper was washed with 0.5% phosphoric acid to remove nonspecific background and incorporated radioactivity was measured.

### Western blotting

Twenty micrograms of parasite lysates or 50 ng of recombinant proteins were resolved by SDS–PAGE on a 10% gel. After separation, proteins were transferred to a nitrocellulose membrane. Membranes were blocked with 5% skimmed milk solution in Tris buffer-saline (pH 7.4) containing 1% Tween-20. Then membranes were probed with either structural CDPK1 antibody (1:1,000 dilution), pS64-phospho-specific CDPK1 antibody (1:1,000) or monoclonal HA antibody (1:1,000). HRP-tagged rat or rabbit secondary antibody was used at 1:2,000 dilution. Blots were developed by enhanced chemiluminescence reagent and fluorograms were collected.

EBA-175 antibody (1:1000) used was an in house antibody generated in the Tobin laboratory against recombinant EBA-175 region III-IV.

### Invasion assay with live merozoites

Merozoites used in the invasion assay were purified as described earlier^57^. Briefly, 3D7 or *Pf*PKG_T618Q_ parasites were synchronized by two sorbitol treatments in the same cycle. Schizonts from 120 ml parasite culture with 4% haematocrit and 2–4% parasitemia were purified on a MACS column. Purified schizonts were treated with 10 μM N-(*trans*-Epoxysuccinyl)-L-leucine 4-guanidinobutylamide (E-64) in complete RPMI medium for 4 h and then schizonts were passed through an Acrodisc 1.2 μm pore membrane syringe filter (Pall Corporation) to collect free merozoites. Free merozoites were added to complete RPMI 1640 medium containing RBC and either 2 μM Compound 2 or the vehicle (dimethyl sulphoxide), then parasites were allowed to invade RBC and grow for 24 h under standard culturing conditions. In one well, Compound 2 was added 1 h post invasion and parasites were allowed to develop to the 24-h time point. Smears were made, stained with Giemsa stain and the percentage of infected RBC was counted by microscopy. The experiment was done three times with duplicate wells for each condition.

### Gel-filtration on Superdex 200

*Pf*CDPK1-HA or *Pf*PKG_T618Q_ parasites were cultured in complete RPMI medium until late schizont stage and harvested on a MACS column. Purified schizonts were resuspended in the same medium, split into two equal aliquots and incubated in the presence of either 10 μM E-64 and 2 μM Compound 2 or 10 μM E-64 and vehicle alone, for 3 h at 37 °C. Following the treatment, samples were incubated on ice with 0.1% saponin in PBS supplemented with protease and phosphatase inhibitors (Roche), the cells were harvested by centrifugation and cell pellets washed twice with TEG buffer (50 mM Tris-HCl, 0.5 mM EDTA, 5% β-Glycerol phosphate, pH 7.4,supplemented with protease and phosphatase inhibitors). The parasite pellets were lysed in TEG buffer (plus protease and phosphatase inhibitors) in the presence of 0.1% NP-40 for 10 min on ice followed by 4 × 10 s sonication. Samples were cleared by centrifugation at 20,000*g* for 3 min and the supernatant fraction was collected and centrifuged again at 20,000*g* for 3 min to remove any further insoluble material. Protein concentration in the soluble lysate was in the range of 2.5–3.0 mg per ml in a final volume of ∼0.5 ml.

This sample (300–350 μl) was then loaded onto a Superdex 200 column (GE Healthcare, 10 × 300 mm) equilibrated with running buffer (50 mM Tris-HCl, 0.15 M NaCl, 20 mM β-glycerol phosphate, pH7.4) and run at a flow rate of 50 μl per min at 4 °C on an AKTA Purifier (Amersham Pharmacia Biotech). Fractions (1 ml) were collected and analysed by western blot—probed with antibodies against the HA-tag or the structural CDPK-antibody.

### Preparation of *P. falciparum* proteins for mass spectrometry

Late-stage schizonts (segmented) were harvested on a MACS CS column (Miltenyi Biotec) in complete RPMI 1640 medium. The parasites were split into two equal aliquots and incubated in the presence or absence of Compound 2 (2 μM) for 1 h at 37 °C in complete RPMI 1640 medium. Following incubation, the parasites were pelleted by centrifugation and resuspended in 0.1% (w/v) saponin in PBS supplemented with protease and phosphatase inhibitors (Roche) and incubated 10 min at 4 °C. The saponin-treated parasites were collected by centrifugation and then lysed with TEG buffer (50 mM Tris-HCl, 0.5 mM EDTA, 5% β-glycerol phosphate, 1% NP-40, pH 7.4) containing protease and phosphatase inhibitors (Roche) on ice for 10 min. Samples were centrifuged at 20,000*g* for 3 min, and the supernatant collected. The pellet was resuspended in TEG buffer without NP-40 and sonicated 3 × 15 s on ice and then centrifuged at 20,000*g* for 3 min. The two supernatant fractions were then pooled. Protein concentration in the final lysates was in the range of 2–4 mg ml^−1^ in a total volume of 0.45–0.5 ml depending on the initial number of cells.

### Tryptic digestion and TMT labelling of parasite proteins

Parasite lysates were diluted in TE buffer (10 mM Tris, 5 mM EDTA, 20 mM β-glycerol phosphate, pH 7.4) to give a final measure of 0.6–0.7 mg protein in 1 ml total volume. This was then incubated at 37 °C for 30 min in the presence 10 mM DTT and 0.1% SDS. Iodoacetamide (1 M solution) was then added to a final concentration of 100 mM and incubation was continued for another 1 h at room temperature in the dark. Protein was precipitated on ice by addition of 100% trichloroacetic acid (at a ratio of 1:4) to the samples and harvested by centrifugation for 3 min at 2,000*g* followed by two gentle washes without disturbing the pellets. The protein pellets were sonicated in 50 mM TEAB buffer (triethylammonium bicarbonate, pH 8.5) and digested with trypsin (Promega) at a ratio of 1:20 (w/w) trypsin to protein content, overnight at 37 °C on a rotating platform.

For tandem mass tag labelling (TMT labelling), completely digested samples were concentrated to about 250–300 μl in a Speedvac centrifuge and labelled with 6-plex TMT reagents (Thermo scientific) following the manufacturer's instructions. TMT-labelled samples were pooled and concentrated to a volume of 750 μl in a Speedvac centrifuge, and then mixed with 250 μl of 100% acetonitrile (ACN) and diluted with 10 mM TEAB, pH 8.0 to a final volume of 3 ml. The sample was loaded onto a Resource Q anion-exchange column (1 ml bed volume, GE Healthcare) equilibrated with 10 mM TEAB, pH 8.0. Peptides were eluted by a linear gradient of 0–1 M NaCl in 10 mM TEAB, pH 7.0, collecting 1 ml fractions. The fractions associated with the flow-through (fractions 2–4) were combined and wash fractions (fractions 5–7) were combined. The flow-through and wash fractions were then concentrated on a speedvac to 1 ml total volume for each. The combined flow-through and wash fractions together with each of the gradient eluate fractions (fractions 10–16) were prepared for immobilized metal affinity chromatography (IMAC) and titanium dioxide (TiO_2_) enrichment of phosphopeptides.

### IMAC enrichment of phosphopeptides for mass spectrometry

Fractions from the anion exchange column step were adjusted to pH 2.5–3.0 by adding 50% trifluoroacetic acid (TFA). PHOS-Select beads (Sigma) were equilibrated with loading/wash buffer (0.25 M acetic acid, 30% acetonitrile) and 50 μl of 50% slurry was added to each fraction, followed by rotating at room temperature for 1–2 h. After incubation with IMAC beads, the samples were transferred onto Mobicol ‘Classic' spin columns (2B Scientific Ltd), centrifuged for 30 s at 1,000*g*, and the flow-through (filtrate) collected and used for further enrichment on TiO_2_ beads as described below. The IMAC beads were washed twice with 200 μl of loading/wash solution, once with 200 μl of HPLC grade water and eluted twice with 100 μl of eluting solution composed of 22 μl ammonia solution (Fisher Chemical, 35% stock), 300 μl acetonitrile to a total volume of 1 ml with water. Eluates were concentrated to a small volume (15–20 μl) on a Speedvac centrifuge and submitted for mass spectrometry analysis.

### TiO_2_ enrichment of phosphopeptides for mass spectrometry

The filtrates from the PHOS-Select-treated samples were used for further phosphopeptide enrichment essentially as described in ref. 58. Briefly, each sample was treated with 5 mg of TiO_2_ beads (GL Sciences Inc) in the presence of 30 mg 2,5-dihydroxybenzoic acid for 1–2 h on a rotating platform at room temperature. Following treatment, the samples were filtered on Mobicol ‘Classic' spin columns, then the beads were washed once with 200 μl of 30% ACN, 0.5% TFA and once with the same volume of 80% ACN, 0.1% TFA by centrifugation for 30 s at 1,000*g*. Phosphopeptides bound to the TiO_2_ beads were eluted twice with 100 μl of 15% ammonia solution. Eluates were concentrated to a small volume (15–20 μl) and submitted for mass spectrometry analysis.

### Mass spectrometry and data processing

LC-MS/MS was carried out using an RSLCnano HPLC system (Dionex) and an LTQ-Orbitrap-Velos mass spectrometer (Thermo Scientific). Samples were loaded at high-flow rate onto a reverse-phase trap column (0.3 mm i.d. × 1 mm), containing 5 μm C18 300 Å Acclaim PepMap medium (Dionex) maintained at a temperature of 37 °C. The loading buffer was 0.1% formic acid/0.05% TFA/2% ACN. Peptides were eluted from the column at a flow rate of 0.3 μl min^−1^ and passed through a reverse-phase PicoFrit capillary column (75 μm i.d. × 400 mm) containing Symmetry C18 100 Å medium (Waters) that was packed in-house using a high-pressure device (Proxeon Biosystems). Peptides were eluted over a period of 4 h, with the output of the column sprayed directly into the nanospray ion source of the LTQ-Orbitrap-Velos mass spectrometer.

The LTQ-Orbitrap-Velos mass spectrometer was set to acquire a 1 microscan Fourier transform mass spectrometer (FTMS) scan event at 60,000 resolution over the *m*/*z* range 300–2,000 Da in positive ion mode. The maximum injection time for MS was 500 ms and the AGC target setting was 1e^6^. Accurate calibration of the FTMS scan was achieved using a background ion lock mass for C_6_H_10_O_14_S_3_ (401.922718 Da). Subsequently, up to ten data-dependent higher-energy collision dissociation (HCD) MS/MS were triggered from the FTMS scan. The isolation width was 2.0 Da, with normalized collision energy of 42.5. Dynamic exclusion was enabled. The maximum injection time for MS/MS was 250 ms and the AGC target setting was 5e^4^.

The raw data file obtained from each LC-MS/MS acquisition was processed using Proteome Discoverer (version 1.4.0.288, Thermo Scientific), searching each file in turn using Mascot^59^ (version 2.2.04, Matrix Science Ltd.) against the UniProtKB-Swissprot database. The peptide tolerance was set to 10 p.p.m. and the MS/MS tolerance was set to 0.02 Da. Fixed modifications were set as carboxyamidomethyl (C) and variable modifications set as oxidation (M) and phosphorylation (S/T/Y). A decoy database search was performed.

The output from Proteome Discoverer was further processed using Scaffold Q+S^60^ (version 4.0.5, Proteome Software). Upon import, the data were searched using X!Tandem^61^ (The Global Proteome Machine Organization). PeptideProphet^62^ and ProteinProphet^63^ (Institute for Systems Biology) probability thresholds of 95% were calculated from the decoy searches and Scaffold was used to calculate an improved 95% peptide and protein probability threshold based on the data from the two different search algorithms.

### Phosphoproteomic data analysis

Plotting the log2 mean fold change of the total peptides from each of the three experiments with wild-type parasites revealed a normal distribution of differences in peptide abundance resulting from the treatment of parasites with Compound 2 (Supplementary Fig. 8). The phospho-peptides that appeared in the lower quartile of the distribution curve in all three wild-type parasite experiments were considered to be downregulated by the treatment with Compound 2.

Next, we examined whether those phosphopeptides considered as downregulated by Compound 2 treatment of wild-type parasites were also downregulated in the Compound 2-treated *Pf*PKG_T618Q_ parasite (note: for a phospho-peptide to be considered downregulated, it had to fall within the lower quartile of the distribution curve in all three wild-type experiments and to be detected in all three of the *Pf*PKG_T618Q_ parasite experiments). This was done using a mixed regression test to determine whether there was a significant difference between the log2 mean fold change of the selected phosphopeptides in the wild-type and *Pf*PKG_T618Q_ parasites. Those phosphopeptides that were downregulated in both the wild-type parasite and in the *Pf*PKG_T618Q_ mutant parasite were considered to result from off-target effects of Compound 2. In contrast, those phosphopeptides that were downregulated in the wild-type parasite samples but were significantly less downregulated in the *Pf*PKG_T618Q_ parasite samples were considered as resulting from on-target effects of Compound 2 and therefore constituted cellular targets of *Pf*PKG.

### Primers

The primers used in this study are listed in Supplementary Table 1.

### Other Data analysis

Where indicated Student's paired *t*-test was used.

## Additional information

**Accession codes:** The mass spectrometry proteomics data have been deposited to the ProteomeXchange Consortium via the PRIDE partner repository with the dataset identifiers PXD002266 and 10.6019/PXD002266.

**How to cite this article:** Alam, M. M. *et al*. Phosphoproteomics reveals malaria parasite Protein Kinase G as a signalling hub regulating egress and invasion. *Nat. Commun.* 6:7285 doi: 10.1038/ncomms8285 (2015).

## Supplementary Material

Supplementary Figures and Tables Supplementary Figures 1-8, Supplementary Table 1.

Supplementary Data 1 Global phosphoproteomic analysis of wild type P. falciparum schizont stage parasites treated with vehicle or compound 2. Shown are the results from three experiments with raw quantification data together with the log2 fold change in the abundance of peptides in the presence of compound 2.

Supplementary Data 2 Global phosphoproteomic analysis of PfPKGT618Q mutant parasite schizont stage treated with vehicle or compound 2. Shown are the results from three experiments with raw quantification data together with the log2 fold change in the abundance of peptides in the presence of compound 2.

Supplementary Data 3 Summary list of 107 phosphorylation sites on 69 phosphoproteins that were defined here as PfPKG cellular targets.

Supplementary Data 4 Matching the 107 phosphorylation sites on 69 phospho-proteins that were defined as PfPKG cellular targets to consensus PKG sites.

Supplementary Movie 1 Z-stack of schizont stage stained with antibodies that detect CDPK1 (red) and phosphorylated CDPK1 (green).

Supplementary Movie 2 Z-stack of merozoite stage stained with antibodies that detect CDPK1 (red) and phosphorylated CDPK1 (green).

Supplementary Movie 3 Z-stack of schizont stage stained with antibodies that detect EBA175 (red) and phosphorylated CDPK1 (green).

Supplementary Movie 4 Z-stack of merozoite stage stained with antibodies that detect EBA175 (red) and phosphorylated CDPK1 (green)

Supplementary Movie 5 Z-stack of schizont stage stained with antibodies that detect TRAMP (red) and phosphorylated CDPK1 (green)

Supplementary Movie 6 Z-stack of merozoite stage stained with antibodies that detect TRAMP (red) and phosphorylated CDPK1 (green)
