# Supplementary material for: Phosphoproteomics reveals malaria parasite Protein Kinase G as a signalling hub regulating egress and invasion
Source: Nat Commun. 2015 Jul 7;6:7285. doi: 10.1038/ncomms8285 (PMC4507021; doi:10.1038/ncomms8285)
Supplement: Supplementary Figures and Tables — Supplementary Figures 1-8, Supplementary Table 1. [file ncomms8285-s1.pdf]

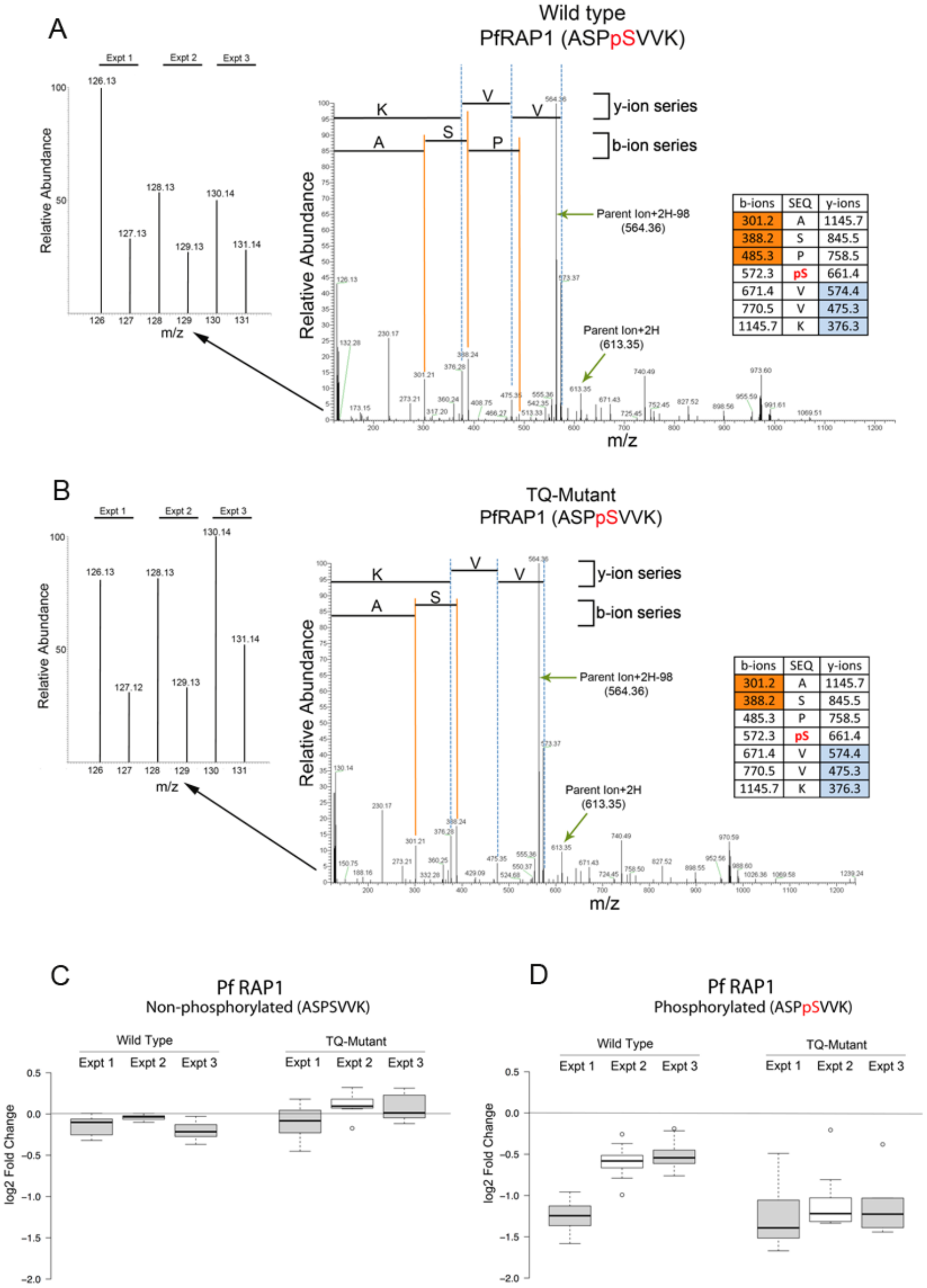

Supplementary Figure 1

Phosphorylation of *Pf*RAP1 – an example of the result of an OFF-target action of Compound 2

(A, B) Representative spectra from the global phosphoproteomic data demonstrating that the phosphorylation of serine-127 on *Pf*RAP1 is down-

regulated following Compound 2 treatment (2 μM, 60 minutes) of both wild type **(A)** and PKG_{T618Q} parasites **(B)**. The inset on the left is a zoom of the spectrum to show the relative abundance of reporter ions from three experiments (126 and 127 from Expt. 1, 128 and 129 from Expt. 2, and 130 and 131 from Expt. 3). The inset on right hand side is the fragmentation table showing the observed b-ions (orange) and y-ions (blue). **(C)** Box plot display of the quantitative changes in abundance of a non-phosphorylated *Pf*RAP1 peptide derived from wild type and PKG_{T618Q} mutant parasites and **(D)** of the phosphopeptide containing serine-127 of *Pf*RAP1.

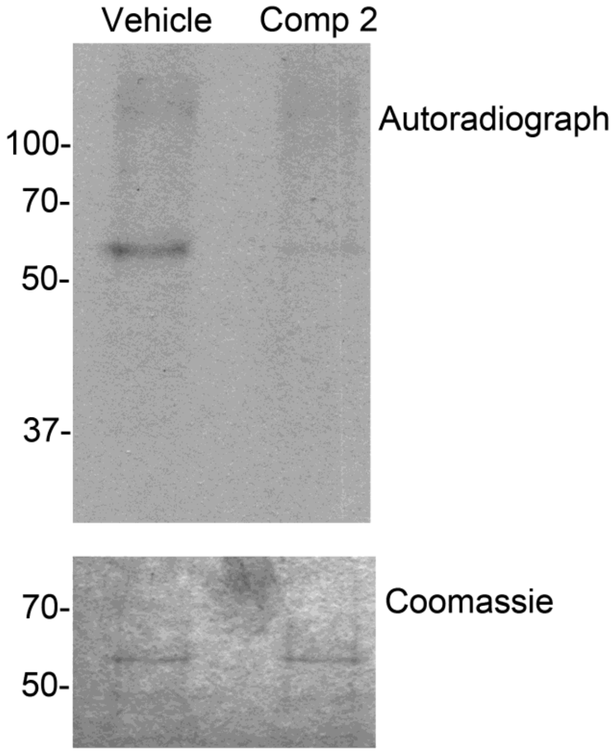

Supplementary Figure 2

Effect of Compound 2 on the phosphorylation of *Pf*CDPK1

Parasites (schizont stage) expressing an HA-tagged *Pf*CDPK1 were metabolically labelled with ^{32}P orthophosphate and treated with vehicle or Compound 2 (2 μM, 40 minutes). The parasites were lysed and *Pf*CDPK1 immunoprecipitated using anti-HA antibodies. Following separation by SDS-PAGE the samples were exposed to autoradiography. Coomassie blue staining of immunoprecipitated *Pf*CDPK1 was used as a loading control. The results shown are representative of three independent experiments.

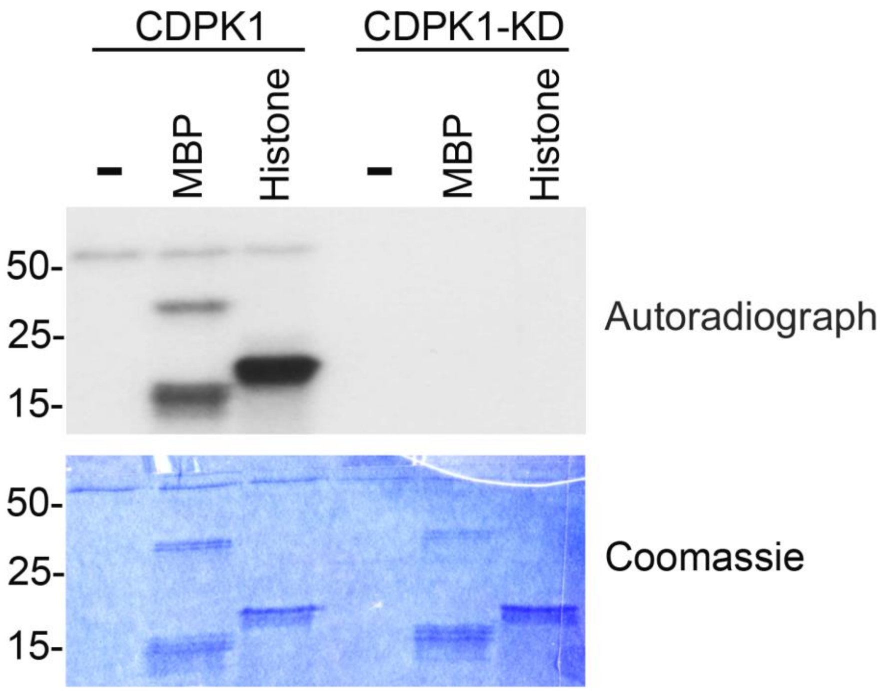

Supplementary Figure 3

Generation of a kinase dead mutant of *Pf*CDPK1

An *in vitro* kinase reaction was carried out using bacterially expressed and purified HIS-tagged wild type *Pf*CDPK1 (1 μg) or a HIS-tagged ‘kinase dead’

mutant of *Pf*CDPK1 in which asparagine-191 was substituted by an aspartate (CDPK1-KD, 1µg), together with myelin basic protein (MBP, 2 µg) or histone (2 µg) as substrates. The reaction was stopped by addition of 2x Laemmli buffer and the proteins resolved by SDS PAGE on a 15% gel. The gel was stained with Coomassie blue, dried and exposed for autoradiography.

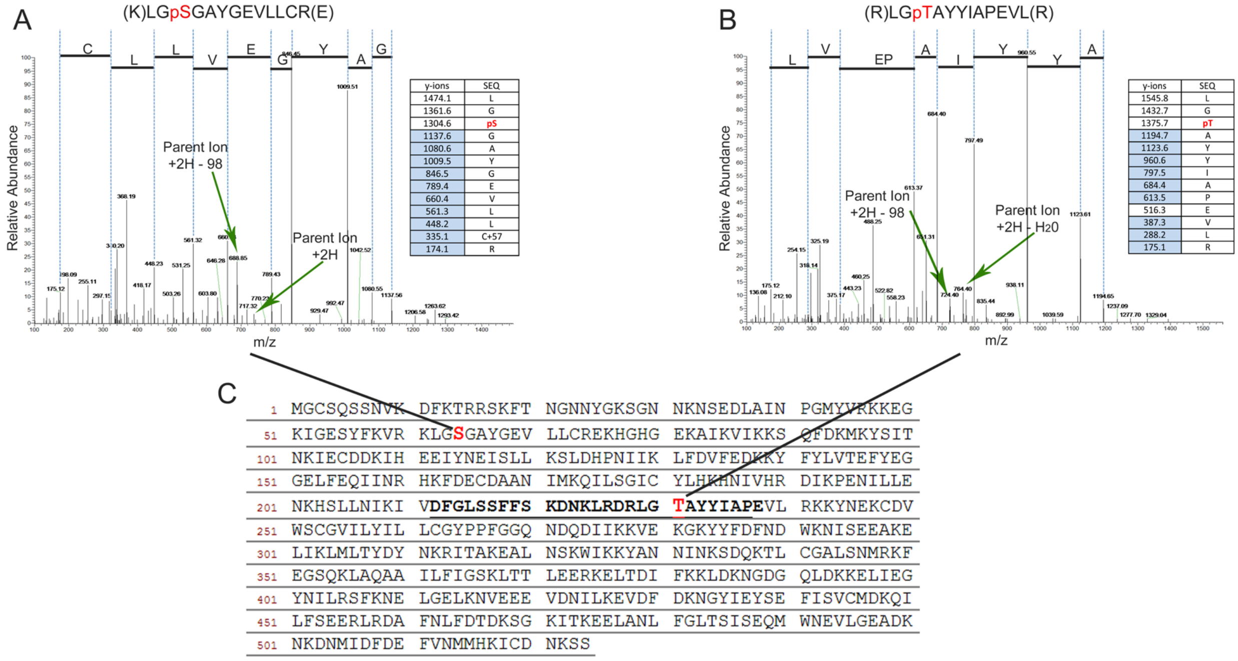

Supplementary Figure 4

Mass spectrometric determination of *Pf*PKG mediated phosphorylation of *Pf*CDPK1 at S64 and T231

Bacterially expressed and purified HIS-tagged CDPK1-KD (1 µg) was used in an *in vitro* kinase assay with recombinant *Pf*PKG (1 µg). The reaction was then analysed by mass spectrometry to determine the sites of phosphorylation on CDPK1-KD. An example of a spectrum demonstrating phosphorylation of **A.** serine-64 and **B.** phosphorylation of threonine-231 is shown. **C.** The

position of S64 and T231 within the primary amino acid sequence of PfCDPK1 is shown (the activation loop is in bold).

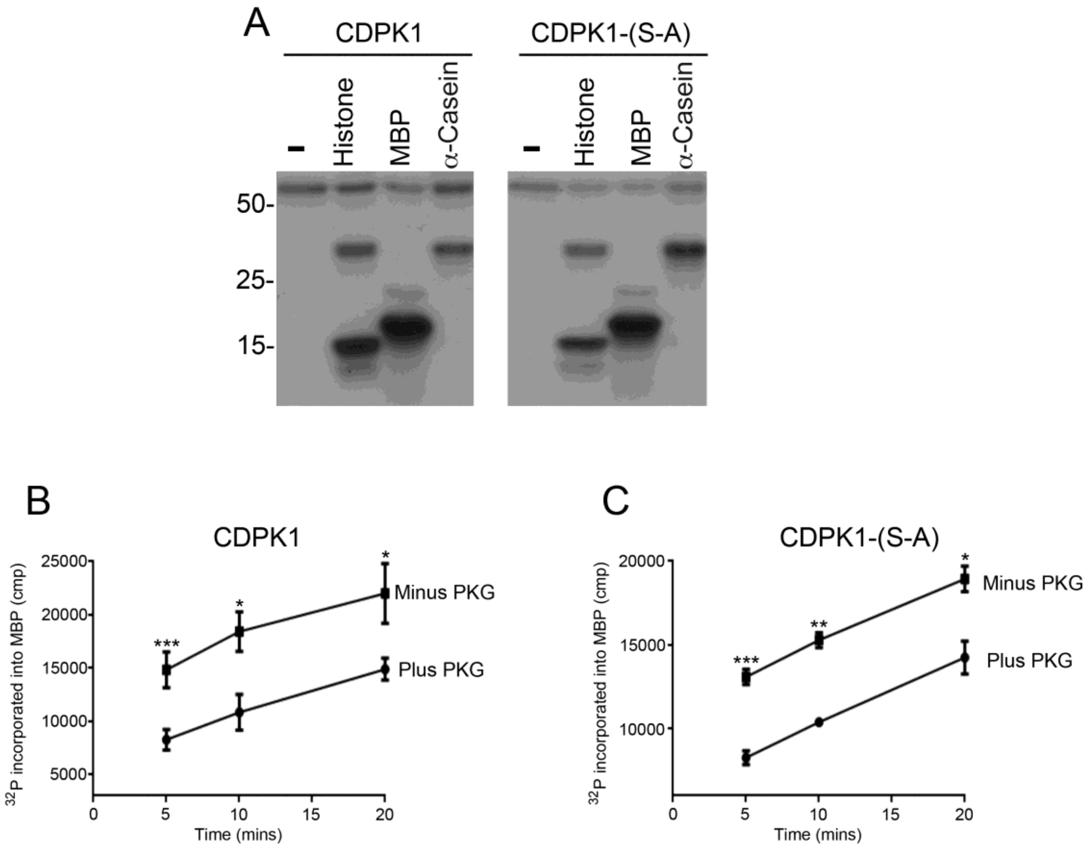

Supplementary Figure 5

Catalytic activity and substrate specificity of *Pf*CDPK1 is not dependent on serine-64 phosphorylation.

(A) Recombinant, purified, HIS-tagged *Pf*CDPK1 (1 μg) and mutant *Pf*CDPK1 (1 μg) in which serine-64 was substituted by alanine (CDPK1-(S-A)) were assayed for protein kinase activity using MBP (2 μg), histone (2 μg)

or α–casein (2 μg) as substrates. The proteins were resolved by SDS-PAGE on a 15% gel and an autoradiograph obtained. The results shown are typical of two experiments. **(B)** The kinase activity of HIS-tagged *Pf*CDPK1 was assayed using MBP as a substrate in the presence or absence of recombinant *Pf*PKG. Following different reaction times, aliquots of the reaction mix were transferred onto P81 nitrocellulose paper and counted for radioactivity. The results are expressed as the mean of three experiments ± S.E.M. **(C)** Same as B except that the mutant CDPK1-(S-A) was used instead of wild type *Pf*CDPK1. Student's paired *t*-test was applied to test statistical significance ***p<0.001, **p<0.01, *p<0.05.

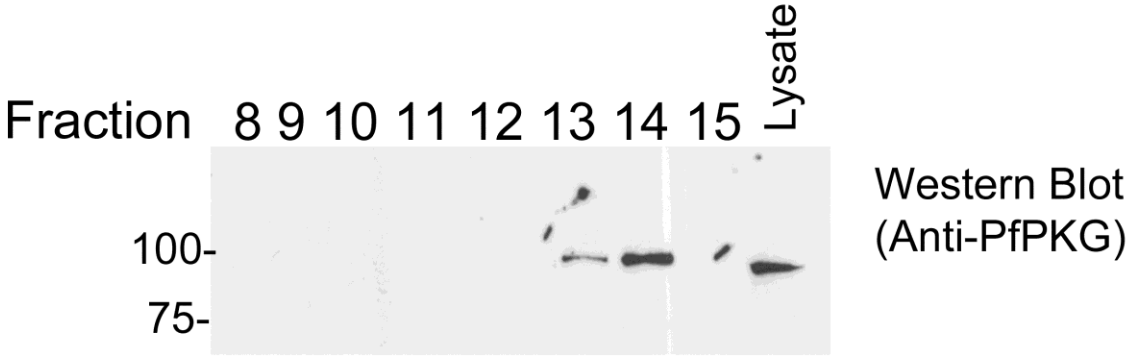

Supplementary Figure 6

***Pf*PKG is not part of the high molecular weight PfCDPK1 complex but exists as monomer.**

Parasite lysate (300μl, approx. 1mg total protein) was fractionated over a Superdex 200 gel filtration column and the fractions probed by Western blot

with an anti-PfPKG antibody. A 15 μl aliquot of lysate was run as a pre-column loading control (lysate). Shown is a representative of three independent experiments.

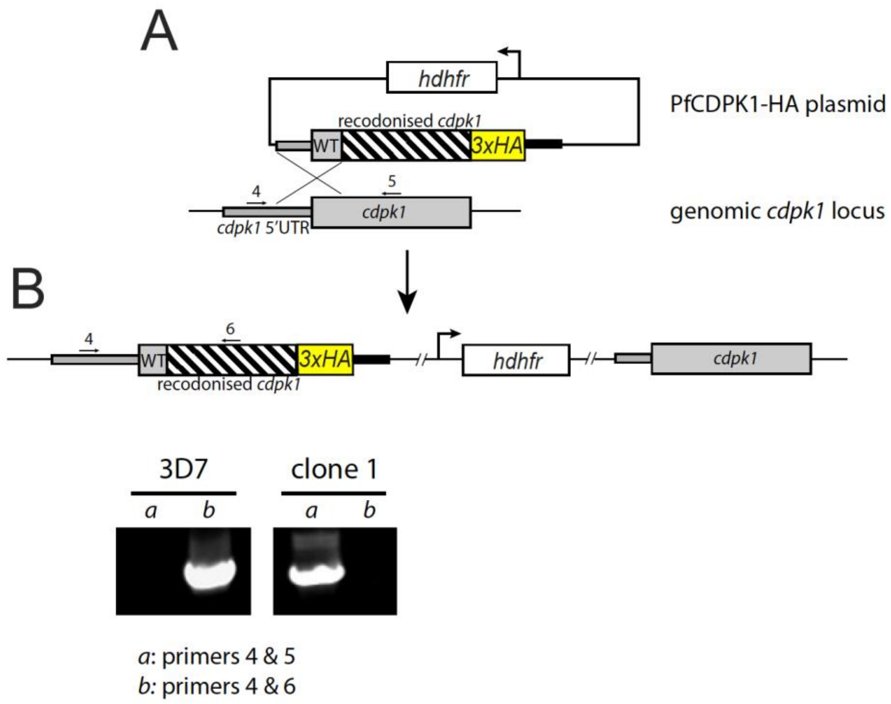

Supplementary Figure 7

Generation of *Pf*CDPK1-HA parasites

(A) Scheme for generating HA-tagged CDPK1 parasites by single crossover recombination. **(B)** Diagnostic PCR to confirm integration of the

plasmid at the *pfcdpk1* locus. Primers 4 and 5 give a product only when correct integration of the plasmid has taken place (lane a), and primers 4 and 6 give a product only from the uninterrupted locus (lane b). For primer sequences see; Primers in supplementary methods.

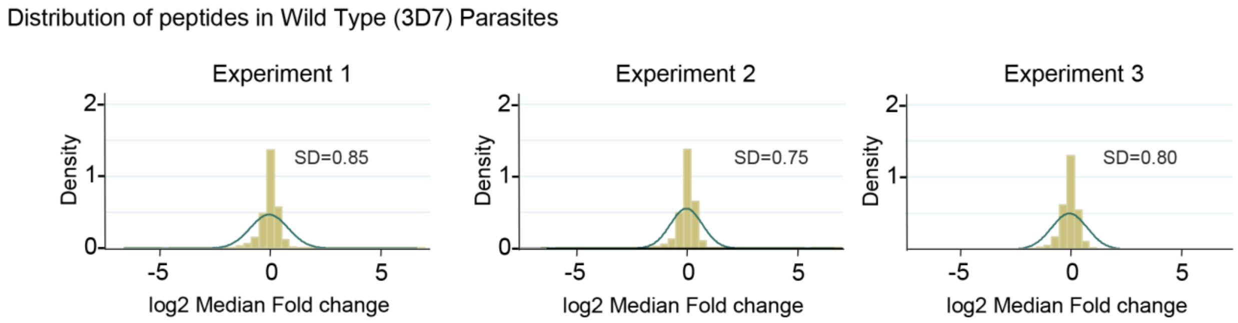

Supplementary Figure 8

Distribution of the changes in the abundance of peptides identified in each of the three global mass spectrometry studies conducted on the wild type *P. falciparum* parasite cultures.

Wild type parasites were treated with vehicle or Compound 2 (2 μM, 60 mins), lysed, trypically digested and the peptides labelled with isobaric tags before the phospho-peptides were enriched. The peptides were then analysed by mass spectrometry and the distribution curves representing changes in the abundance of the peptides plotted. Those phospho-peptides that were seen to be present in the lower quartile of the distribution curves in all three experiments were then compared with the same peptides present in the three global phosphoproteomic experiments conducted on the PKG_{T618Q} mutant

parasites. Those phosphopeptides that were seen to be reduced in both the wild type and mutant parasites were considered to represent the off-target action of Compound 2 and those peptides that were only seen to be reduced in the wild type parasites were the on-target action of Compound 2 and therefore considered as cellular targets for *Pf*PKG.

Supplementary Table 4

PRIMER	SEQUENCE
CDPK1_FL_His_Fwd	AGGAGATATACATATGGGGTGTTCACAAAGTTCAAACG
CDPK1_FL_His_Rev	GAAGTACAGGTTCTCTGAAGATTTATTATCACAAATTTTGTGCATC
CDPK1_FL_GST_Fwd	ATGCGGATCCATGGGGTGTTCACAAAGTTCAAACG
CDPK1_FL_GST_Rev	ATGCGGATCCTTATGAAGATTTATTATCACAAATTTTGTGCATC
CDPK1_D191N_Fwd	CATATGTTATTTACATAAACATAATATTGTACATCGAAATATTAAACCAGAAAATATTTTATTAGAAAATAAACA
CDPK1_D191N_Rev	TGTTTATTTTCTAATAAAATATTTTCTGGTTTAATATTTCGATGTACAATATTATGTTTATGTAAATAACATATG
CDPK1_S64A_fwd	CTATTTCAAAGTTCGCAAATTAGGTGCCGGTGCGTACGGA
CDPK1_S64A_Rev	TCCGTACGCACCGGCACCTAATTTGCGAACTTTGAAATAG
PRIMER 1	ACACCCCGGGGTATACAACGTATAAGACAAATTACTTTTCTTTC
PRIMER 2	ATATGAATTCGGTTACTAAATAAAAATATTTCTTATCTTCAAAAACATCAAAC
PRIMER 4	GATGGTGGCACTTGCCTTTTTGAGG
PRIMER 5	CCCAATCTGTCCCTTAGCTTGTTGTC
PRIMER 6	CTGGTTTAATATCTCGATGTACAATATTATGTTTATG

Supplementary Table 1

List of PCR primers used in this study.
